# Analysis of inter-country input-output table based on citation network: How to measure the competition and collaboration between industrial sectors on the global value chain

**DOI:** 10.1371/journal.pone.0184055

**Published:** 2017-09-05

**Authors:** Lizhi Xing

**Affiliations:** School of Economics and Management, Beijing University of Technology, Beijing, China; University of Rijeka, CROATIA

## Abstract

The input-output table is comprehensive and detailed in describing the national economic system with complex economic relationships, which embodies information of supply and demand among industrial sectors. This paper aims to scale the degree of competition/collaboration on the global value chain from the perspective of econophysics. Global Industrial Strongest Relevant Network models were established by extracting the strongest and most immediate industrial relevance in the global economic system with inter-country input-output tables and then transformed into Global Industrial Resource Competition Network/Global Industrial Production Collaboration Network models embodying the competitive/collaborative relationships based on bibliographic coupling/co-citation approach. Three indicators well suited for these two kinds of weighted and non-directed networks with self-loops were introduced, including unit weight for competitive/collaborative power, disparity in the weight for competitive/collaborative amplitude and weighted clustering coefficient for competitive/collaborative intensity. Finally, these models and indicators were further applied to empirically analyze the function of sectors in the latest World Input-Output Database, to reveal inter-sector competitive/collaborative status during the economic globalization.

## Introduction

This paper mainly focuses on how industrial sectors compete for their production resources from mutual providers and how they collaborate to facilitate the production for mutual consumers. **Input-Output (IO)** data were adopted to establish industrial complex network models, in order to carry out detailed analyses on the **Global Value Chain (GVC)** from the perspective of econophysics. Followed is review of relevant literature of the research.

### Industrial complex network

The industrial complex network is a kind of social network, in which product sectors are intricately interrelated by the products and services they both provide and consume. The sectors are taken as nodes and the various economic relationships among them as edges in the network, which can bring the various economic issues according under analysis.

In last two decades, a large number of theoretical and empirical researches on industrial economics based on a complex network have been accomplished in areas of industrial development, structure, association, organization and policy-making. Chmiel, et al. established networks of companies and branches in Poland through bipartite graph theory [1]. Similarly, Inoue, et al. investigated a Japan’s patent network focusing on its spatial characteristics based on the same theory [2]. Chang, et al. proposed that the degree distribution of nodes in a projected sub-network follows the drift power-law in general cooperation as well as in competition networks [3]. Liu, et al. studied the development of China’s high-tech park using complex networks, and constructed the network of China’s top 100 electronic and IT companies as the basic model [4]. Hou, et al. extended their research fields from monopoly market to macro-reality market to build competitive complex network model targeting logistics enterprises [5]. Li, et al. established a global nuclear power plant network based on priority queuing network model and simulated its numerical characteristics to reflect its evolution [6].Yao, et al. modeled the directed weighted competitive pressure network and made a simulation analysis on the rivalry spread effect on it [7].Upper analyzed the mechanisms of contagion in banking and financial networks [8]. Hu, et al. calculated economic distance matrices based on annual GDP of nine economic sectors from 1995–2010 in 31 Chinese provinces and autonomous regions, and built up spatial economic networks via threshold and minimal spanning tree [9].

Scholars have created various complex network models to describe inter-organization competition and collaboration analyzing diverse economic phenomena. But the early works are prone to use binary approaches, i.e., they introduced non-weighted and non-directed network models similar to the simple physical ones, with less to be known or much to be neglected on the mechanism of informational, material and capital flows between economic entities manifested in their dependencies. However, the literature on industrial complex networks are growing rapidly, and increasing number of scholars focus on sophisticated topological structure of economic system based on weighted and directed graphs. In the meanwhile, relevant empirical researches incorporate more economic issues, such as investment stocks [10], inter-bank connection [11], innovation [12], ownership [13], systemic risk [14], information flow [15], environmental capacity [16], etc.

### Input-output network

From an empirical perspective, a handful of studies has characterized the structure of IO networks to better understand the topology of inter-industry dependencies and their repercussions on the industrial economics. For instance, Blöchl, et al. adopted structural analysis database (STAN) of Organization for Economic Co-operation and Development (OECD) to establish 37 countries’ IO networks. They derived two indicators for weighted and directed network with self-loops, which are, random walk centrality to reveal the most immediately affected nodes by a shock based on Freeman’s closeness centrality, and counting betweenness to identify the most accumulatively affected nodes based on Newman’ random walk betweenness [17]. Kagawa, et al. proposed an optimal combinatorial method to find industries with large CO2 emissions through industrial relations based on IO table, depicting environmentally important industrial clusters in Japanese automobile supply chain [18]. McNerney, et al. studied the structure of inter-industry relationships using networks of capital flows between industries in 20 national economies, and found these networks vary around a typical structure characterized by a Weibull link weight distribution [19]. Martha, et al. investigated how economic shocks propagate and amplify through the IO network connecting industrial sectors in developed economies [20].

With the development of IO databases, related researches are no longer limited to independent national systems but extended to multi-regional even global systems, while most of them adopt World Input-Output Database (WIOD) as the data source. For instance, Ando measured the importance of industrial sectors under the impact of U.S. gross output with the global IO model [21]. Li, et al. introduced a network-based methodological framework for quantifying the importance of each industry and country according the gross damage that may result due to their failure [22]. Zhu et al. introduced a network-based measure on node similarity to compare the GVC between any pair of countries for each sector and year available in the WIOD [23]. Cerina, et al. analyzed the subgraph structure and dynamics attributions of global network with community detection techniques, pinpointing the key industries and economic entities with PageRank centrality and community coreness [24]. Grazzini and Spelta set up the cost effect index to testify the robustness of global IO network and the interdependency of intermediate inputs in production [25]. Amador and Cabral applied visualizing tools and measures for network analysis on value-added trade flows in order to understand the nature and dynamics of the GVC [26]. Xing, et al. analyzed the diffusion effect of industrial sectors with complex network model under the perspective of econophysics [27], furthermore, they quantified the individual impact of each country on the GVC based on biased random walk process [28].

The existing researches mainly mine the IO data from different aspects as an econophysics context implied in the form of networks but restricted to statically analyzing endogenous variables ignoring the process of fining and refining of variables to maintain equilibrium, let alone providing measurements and advises on optimal control of the evolution tendency of industrial structures.

### Global value chain

In recent years, considerable works have been done on analyzing economic globalization. Compared with firm surveys and fine industrial classification of trade, IO tables enjoy more feasibility in measuring both standard and vertical trades. With the availability and utilization of global IO databases, especially **Inter-Country Input-Output (ICIO)** tables, it is possible to construct quantitative indicators to assess what degree of impact a particular sector in a country has made on the GVC, because it better captures the international source and use of intermediate goods than any previous databases. As results, a large number of researches have proposed distinct approaches to the measurement of sectors’ functions or status.

Hummels, et al. focused on the use of imported inputs in producing goods to be exported and named it vertical specialization, which is the first empirical measure of participation in vertically specialized trade [29]. Antràs, et al. derived two distinct approaches to measure industry upstreamness and prove their significant impact on trade flows [30]. Fally provided quantitative analyses on the average length of production chains, which tells the number of stages required for production and the number of stages between production and final consumption [31]. Then, he and Hillberry extended the empirical research from across plants to across borders by employing the 4-dimensional input-output tables from Institute of Developing Economies Japan External Trade Organization (IDE-JETRO) [32]. Johnson and Noguera combined input-output and bilateral trade data to quantify cross-border production linkages and computed bilateral trade in value added [33]. Koopman, et al. integrated all previous measures on vertical specialization and value-added trade to adjust for the back-and-forth trade of intermediates across multiple borders, and presented GVC position and participation indices to gauge the extent to which a country-sector is involved in the global production chain. In order to empirically conduct gross export decomposition, they constructed a global ICIO based mainly on version 7 of the GTAP database [34]. Furthermore, with the update and improvement of ICIO databases, all of the research frameworks mentioned above can be applied to generate a time series decomposition of gross trade flows into value-added components.

Studies on the GVC have turned to take advantage of ICIO databases. However, basic ICIO databases are unable to distinguish imported intermediate from final goods in bilateral trade flows, and more importantly, they overlook the fact that heterogeneity generally exits in economic endowment, geographical location, development stage, industrial structure etc. at the domestic and regional levels. Thus, more and more scholars are paying attention on how global production is fragmented and extended internationally or domestically. To be specific, domestic linkages are measured via endogenously embedding a target country’s domestic **Inter-Regional IO (IRIO)** tables into the ICIO ones, building up **Regionally-Extended Inter-Country Input-Output (REXICIO)** tables [35–36]. Surprisingly, this emerging framework precisely follows the concept of super-network in the field of econophysics. However, it is hard to make an effective analysis on the GVC due to the time-validity of IRIO, e.g., China’s latest IRIO was released in 2007 while Japan’s in 2005 [37].

## Data

Beyond all question, IO table as a quantitative technique of economic analysis presents the dependencies between different branches of a national or regional economy in details. This paper adopts ICIO data to present the operating mechanism of a global economic system, thus it is necessary to review the superiority and availability of IO data.

### Advantage of IO table

The IO model is a technique that quantifies interdependency in interconnected economic systems. Wassily Leontief first introduced the IO model in 1951 [38], for which he received the Nobel Prize in Economics in 1973. It can be used to study the effect of consumption shocks on interdependent economic system [39]. IO analysis is the study of quantitative relations between the output levels of the various sectors of an economy, a practical tool for national accounting and planning. Neoclassical economics focusses on the pure theory of the price mechanism, equilibrating supply and demand in free market economies [40].

As the basic status and structure of IO data, its property of being in the format of check board enables it to reproduce the movements of products or services within the whole economic system, including both production consumption and distributive utilization, which are actually the formation and distribution of values respectively. The dual identities of each sector as the producer and consumer at the same time, demand it not only to produce and distribute providing outputs to the other sectors but also to consume inputs from other sectors to accomplish its own fabrication. This is indeed the inner identity proposed by Karl Marx.

The sectors in IO table could be regarded as nodes while inter-sector value stream contributes to weighted and directed edges in the construction of network model. In consideration of both availability and authority, IO table is definitely the priority-first data source to establish mathematics model, e.g., it can show flows of final and intermediate goods and services. In addition, it is provided in a matrix, which can be directly or with minor modification adopted as adjacency matrix in establishing weighted and directed networks.

IO network model is able to reveal the mechanism of creation, distribution, transfer and value-addition of value in economic system. The common IO analysis adopts the direct consumption and complete consumption coefficients matrices to show the direct and indirect technical-economic relationships among industrial sectors, before using influence and reaction coefficients to measure the pulling effect and demand intensity of one sector on the other. But no existing research has brought researches on the competitive/collaborative relationships to the level of industries, for there lies the difficulty of distinguishing the functional roles of any industry in outputting or consuming the intermediates. For this reason, this paper aims at consolidating the IO analysis and network-based approach to find a way out.

### Avaliable ICIO database

The advent of ICIO databases has made it theoretically and empirically possible to have fine analysis on GVC, which is composed of abundant international and domestic industrial value chains, because ICIO tables provide globally consistent bilateral trade flows and allow comparison of production networks in different regions. The layout of a normal ICIO table is shown in Table 1, and we took the region of inter-country and inter-industry use and supply as modeling data source in this paper, i.e., the yellow part of the table, in order to depict the transferring process of intermediate goods under the perspective of complex network.

**Table 1:** Layout of ICIO table.

		Intermediate Use			Final Use			Total Use
		Country 1	…	Country M	Country 1	…	Country M	
Intermediate Input	Country 1							
	…							
	Country M							
Value Added								
Total Output								

There are seven main ICIO databases available presently: World Input-Output Database (WIOD), OECD-WTO Database on Trade in Value-Added (TiVA), Eora Multi-Region Input-Output Table Database (MRIO), Global Trade Analysis Program (GTAP), Asian International Input-Output (AIO), Asian Development Bank Multi-Regional Input-Output Tables (ADB-MRIO) and Externality Data and Input-Output Tools for Policy Analysis (EXIOPOL).

As a sort of value-type IO table, WIOD was chosen as the underlying database. The latest version of WIOD was released in 2016 (WIOD2016R, for short), covering 43 countries and the rest of the world for the period 2000–2014. Data for 56 sectors are classified according to the International Standard Industrial Classification revision 4 (names and abbreviations of countries and sectors in WIOD2016R are in S1 File) [41]. These tables have been constructed in a clear conceptual framework on the basis of officially published IO tables in conjunction with national and international trade [42]. Since publication, it has been proved very useful in analyses on global chain trade [43], domestic value-added content of gross exports [44], effects of trade policies [45], offshoring on labor demand [46] and policy-oriented studies [47–48]. It is foreseeable that there will be more applications of WIOD in multiple research fields.

## Methodology

Taking inspiration from citation network, competitive relationships can be abstracted from IO data via bibliographic coupling approach, and collaborative relationships via co-citation approach, which constitute the research framework of this paper. However, citation network used to be restricted to the studies of scientific collaboration. Thus, close attention should be paid on not only the derivation of their generalized forms but also their potential application in IO network.

### Conception of citation network

**Fig 1:**
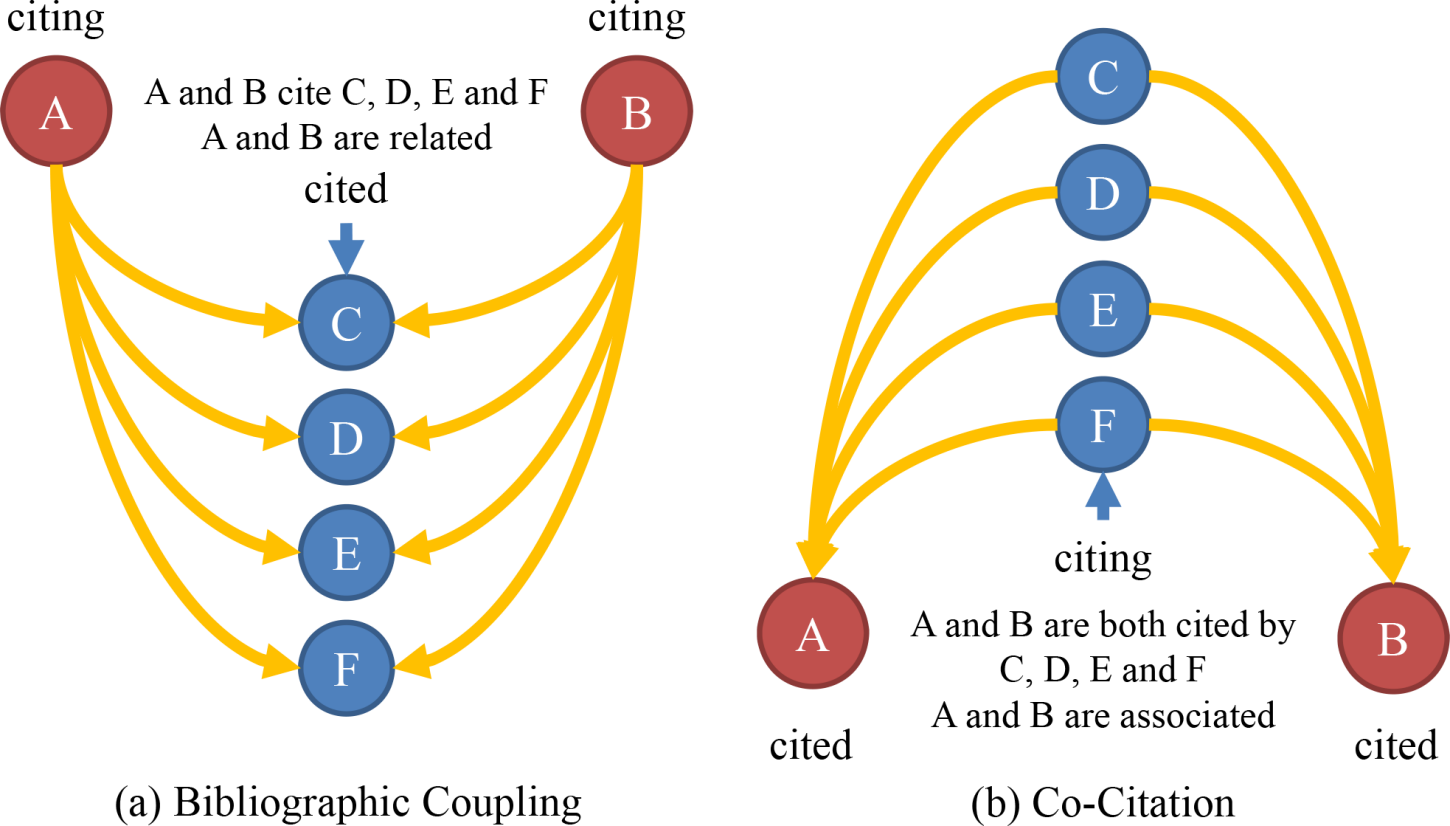
Bibliographic coupling and co-citation.

In citation network, bibliographic coupling and co-citation are two means to measure semantic similarity of documents which are in citation relationships. In details, documents are bibliographically coupled when different authors cite one or more documents in common, while co-citation is based primarily on identifying documents of high-cited documents [49]. Their basically format differences are shown in Fig 1.

The differences between bibliographic coupling and co-citation approaches are as follows:

1. Bibliographic coupling reflects the relationships between two documents citing the same source s, which can only be established by two authors together. However, co-citation reflects the relationships between two documents cited by a third part, and many authors contribute to this process.
2. The degree of bibliographic coupling is fixed, but that of co-citation maybe change at any moment because there is the possibility that emerging documents would cite the same source.
3. Bibliographic coupling measures the constant and long-term inter-document relationships, and co-citation refers to varying and temporary type. Therefore, static and dynamic structural models are respectively based on these two methods in citation network analysis in general.
4. Bibliographic coupling is retrospective whereas co-citation is essentially a forward-looking.
5. Co-citation is superior to bibliographic coupling in both revealing the inner relationship between scientific literature and depicting the dynamic structure of scientific development.

Co-citation has got more and more attention than bibliographic coupling in the application. Small first defined co-citation as the frequency of which two documents are cited together by a third document. If a third document cites two documents in common, they are said to be co-cited. The more co-citations two documents receive, the higher their co-citation strength is, and the more likely they are semantically related [50]. Over the decades, researchers have proposed variants or enhancements to the original co-citation approach. For instance, White introduced author co-citation analysis [51]; Gipp and Beel proposed co-citation Proximity Analysis (CPA) and introduced the co-citation Proximity Index (CPI) as an enhancement to the original co-citation concept [52]; Shen and Barabási developed a credit allocation algorithm that captures the coauthors’ contribution to a publication as perceived by the scientific community, reproducing the collective credit allocation of science [53]; Schwarzer, et al. proved that CPA considers the proximity of citations within the full-texts for similarity computation and therefore allows for a better assessment of semantic document similarity than pure co-citation [54]. Bibliographic coupling on the other hand has also huge potential in many fields of researches, with its definition and calculation similar to those of co-citation.

Given that nodes represent documents in a directed network, if both nodes *i* and *j* cite node *k*, i.e., two edges respectively start from node *i* and *j* then reach node *k*, there will be an edge between node *i* and node *j* in a newly formed bibliographic coupling network. By contrast, if node *k* cite node *i* and *j* at the same time, all of the abstract edge coming from node *i* and node *j* constitute the so-called co-citation network.

**Fig 2:**
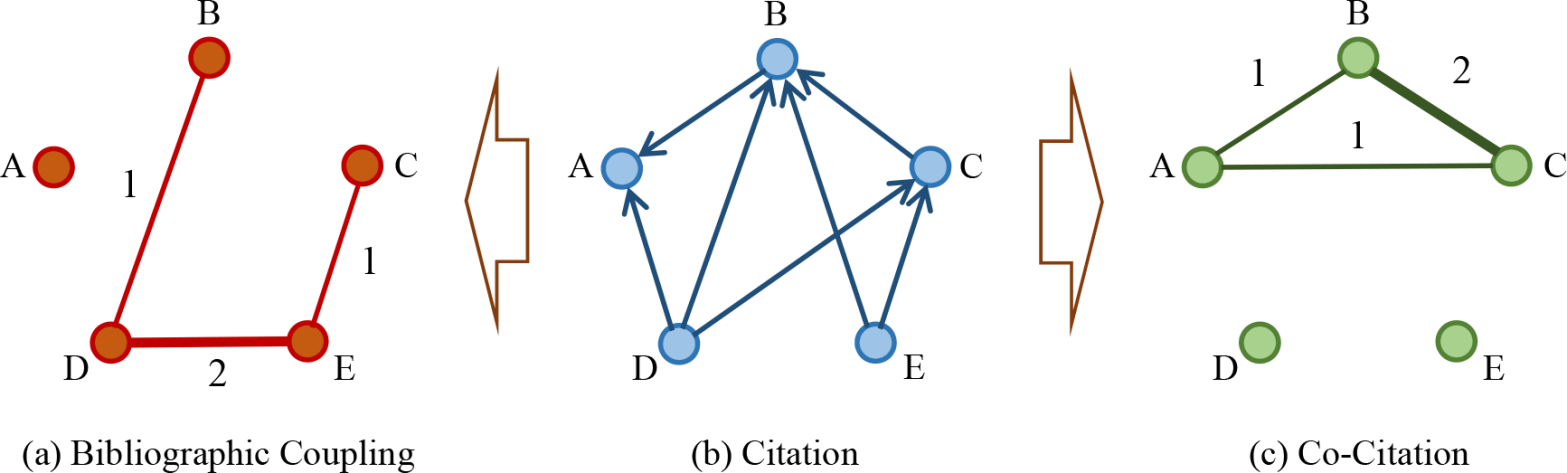
How citation relationship turns to be bibliographic coupling and co-citation relationships.

In these two kinds of networks, directions of edges disappear because there is no need to distinguish the difference existing in two pairs of citation relationship. Thus, the times of two arbitrary nodes are cited by others at the same time is called bibliographic coupling strength, and that of the opposite is called co-citation strength. To better understand these two approaches, an example containing five nodes as well as their two categories of relationships is given in Fig 2.

As shown in Fig 2(B), the citation relationships among five nodes constitutes a directed network. According to the basic bibliographic coupling approach mentioned above, original directed network is transformed into a non-directed one in Fig 2(A), which describes the bibliographic coupling relationships among them. In details, B and D collectively cite A for one time in citation network, so the edge weight between them in bibliographic coupling network is equal to 1; D and E cited B as well as C, i.e., the phenomena of bibliographic coupling happen twice, hence their edge weight is equal to 2; A cites no one, for it is probably the one published earliest, so there is no bibliographic coupling relationship about it. Similarly, co-citation strength can be roughly quantified as shown in Fig 2(C).

### Generalized bibliographic coupling and co-citation

Citation network model has already been applied to numerous researches about collaboration among scholars, and it has huge potential as modeling framework in more fields of complex network. However, there are several important issues needed to be solved before introducing it in other directed networks.

First and foremost, there must be reasonable explanation for the newly formed bibliographic coupling/co-citation network. Bibliographic coupling/co-citation relationships abstracted from citation network represent some kinds of potential relevance, such as academic collaboration, substitution of choices (maybe traffic routes, commodities, strategies, etc.), even potentially competitive/collaborative status. If this approach is feasible, the relationships obtained from all generalized co-cited or co-citing objectives can be further quantitatively measured.

Secondly, the chronological order of research objectives in static network analysis is usually ignored. That is to say, it doesn’t matter that cited nodes appear before citing nodes or not, but it is essential that bibliographic coupling/co-citation network based on nodes’ topological structure of citation is correctly established, which is weighted and with self-loops sometimes. For instance, if there is an out-edge starting from one node and reaching two other nodes, a generalized bibliographic coupling relationship between them occurs in fact.

Thirdly, taking applicability into account, generalized bibliographic coupling and co-citation approaches for weighted and directed networks with self-loops are proposed here. According to the definition of adjacency matrix of directed network, if the node *k* has two out-edges pointing at node *i* and *j*, *a*_*ki*_*a*_*kj*_ = 1, otherwise *a*_*ki*_*a*_*kj*_ = 0, where *a*_*ij*_ is the i-th row and j-th column element of the adjacency matrix of citation network *A* = {*a*_*ij*_}. Under the same conditions, if the node *k* has two in-edges coming from node *i* and *j*, *a*_*ik*_*a*_*jk*_ = 1, otherwise *a*_*ik*_*a*_*jk*_ = 0. Thus, the bibliographic coupling and co-citation strength of node *i* and *j* (*i* ≠ *j*) are respectively as follows:

$$b_{ij}=\sum_{k=1}^{N}a_{ki}a_{kj}$$ (1)

$$c_{ij}=\sum_{k=1}^{N}a_{ik}a_{jk}$$ (2)

where *b*_*ij*_ is the i-th row and j-th column element of the adjacency matrix of bibliographic coupling network, *B* = {*b*_*ij*_}, and *c*_*ij*_ is that of co-citation network, *C* = {*c*_*ij*_}. The diagonal elements of two network are:

$$b_{ii}=\sum_{k=1}^{N}a_{ki}^{2}=\sum_{k=1}^{N}a_{ki}$$ (3)

$$c_{ii}=\sum_{k=1}^{N}a_{ik}^{2}=\sum_{k=1}^{N}a_{ik}$$ (4)

where, *b*_*ii*_ is equal to the degree of node *i* because citation network is usually assumed to be binary, which is namely the number of nodes linked to node *i*, and *c*_*ii*_ is equal to the in-degree of node *i*. In the matrix form, there are:

$$B=A^{T}A$$ (5)

$$C=AA^{T}$$ (6)

**Fig 3:**
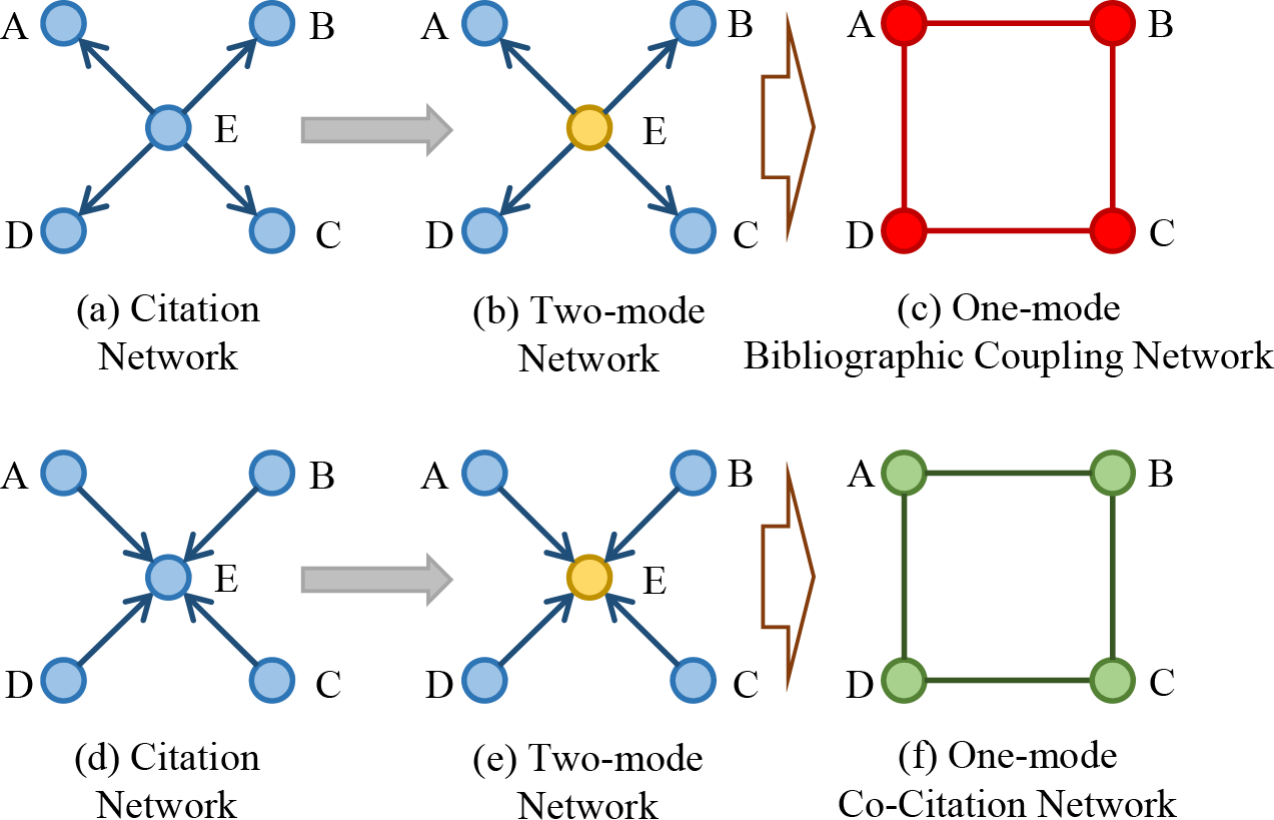
Another view on how citation network is transformed into bibliographic coupling and co-citation networks.

From another perspective, citation network can be treated as a special form of two-mode network in the process of transforming it into bibliographic coupling/co-citation network, as shown in Fig 3.

Say nodes A, B, C and D cite node E simultaneously, can form a citation network, as shown in Fig 3(A). When measuring their bibliographic coupling relationships, node E is differently treated as an individual set to be distinguished from the others. Therefore, these nodes can be divided into two sets temporarily as shown in Fig 3(B). This two-mode network can be transformed into a one-mode network by projection, which is also a bibliographic coupling network, as shown in Fig 3(C). This process works by selecting one set of nodes (A, B, C and D), and linking two nodes if they are connected to the same node of the other set (only E). If there are more “E” for the others (i.e., projection repeats for several times), multiple citation relationships from the two-mode network can be incorporated as edge weights in the bibliographic coupling network. By repeating the same process, the citation network, in which node E cites the other four nodes as shown in Fig 3(D), can also be transformed into a one-mode co-citation network.

Traditionally, the edges in the projected one-mode network do not have weights. However, recent empirical studies on two-mode networks have created kinds of weighted one-mode network by defining the weights as the number of co-occurrences (e.g., the number of events two individuals have co-attended, or the number of documents that two authors had collaborated on) [55]. For instance, Newman extended this procedure working on scientific collaboration networks [56–58]; Padrón, et al. proposed that this method of modeling network makes realistic prediction on the potential competitive or facilitative interactions among species of one set [59].

### Application of citation network in IO analysis

As mentioned above, IO table is good at presenting sophisticated inter-sector dependencies from a global perspective, with which one can perceive how much production resources that sectors obtain from upstream ones. Actually, competition/collaboration occurs when sectors own the same providers/consumers, because all sectors’ products and services outputted to downstream ones are limited. Thus, there is a possibility that inter-sector competition for inputs from upstream sectors, or collaboration on outputs to downstream sectors, can be quantified with IO data with bibliographic coupling/co-citation approach. However, both of them usually ignore the fact that edges in original citation network do not have weights. Therefore, they also need to be revised to fit IO networks with edge weights and self-loops.

**Fig 4:**
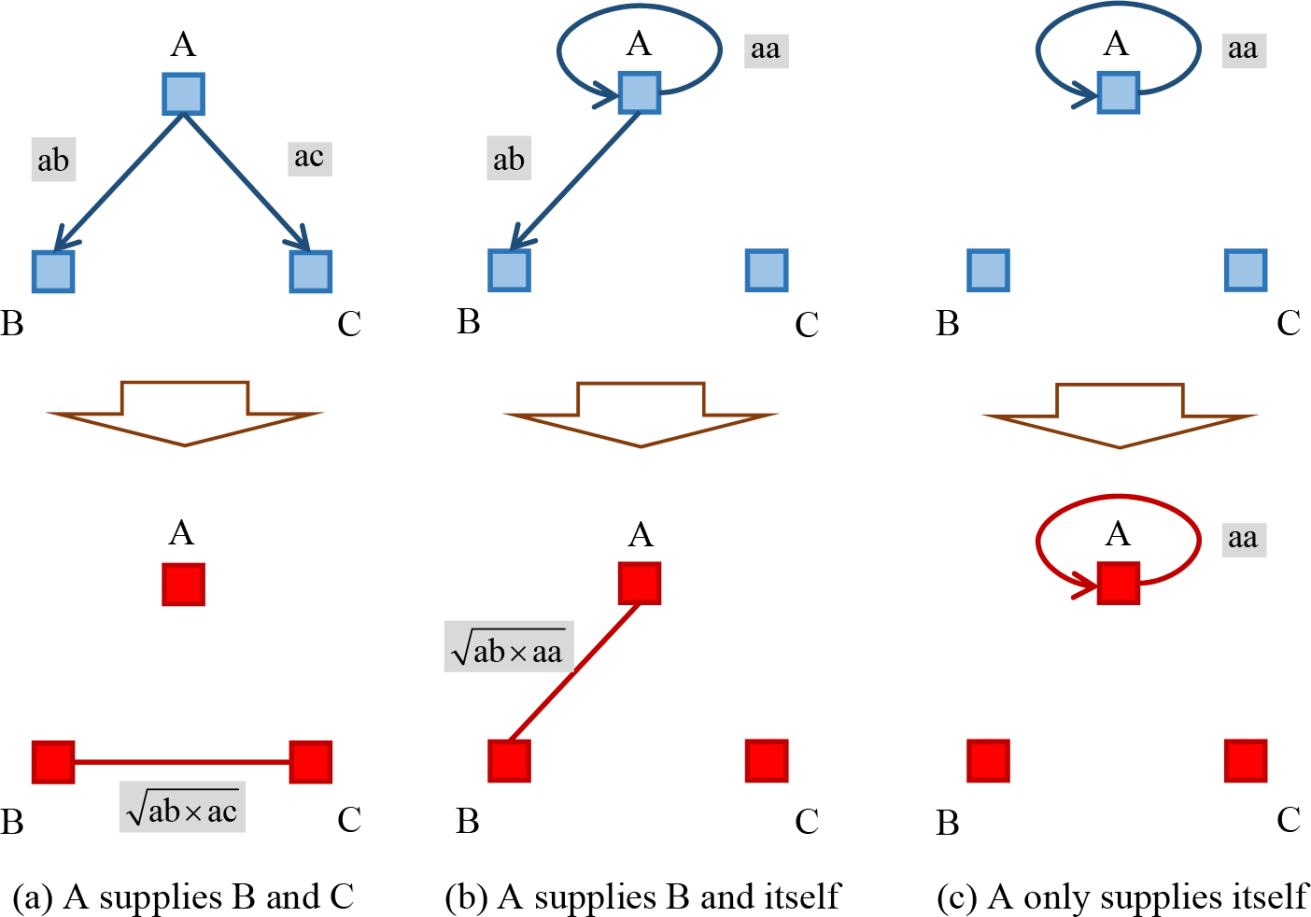
Three situations of transforming IO network with bibliographic coupling approach.

Moreover, there exist three situations of inter-sector competition for production resources as shown in Fig 4A, 4B and 4C represent different industrial sectors respectively, ab and ac represent the IO values between them. aa is especially the self-consumption of A, i.e., the weight on the self-loop.

First of all, if A supplies both B and C with limited production resources as shown in Fig 4(A), and the quantitative degree is measured by the IO value between them, thus competitive relationship occurs between B and C. The more upstream providers B and C have simultaneously, the more fierce competition they will suffer. Single competition strength is here defined as the square root of ab multiplying ac, and multiple competition strength is then equal to the accumulation of single ones when there are more nodes acting as A for B and C.

Secondly, if A supplies B and itself as shown in Fig 4(B), their single competition strength is defined as the square root of ab multiplying aa. However, there could not be any multiple competition between A and B under this circumstance.

Finally, if A only supplies itself as shown in Fig 4(C), its single competition strength is equal to aa, which is its own consumption.

Conclusively, each sector obtains resources from its upstream providers or itself, otherwise the node representing it will be out of the largest connected component of industrial complex network.

**Fig 5:**
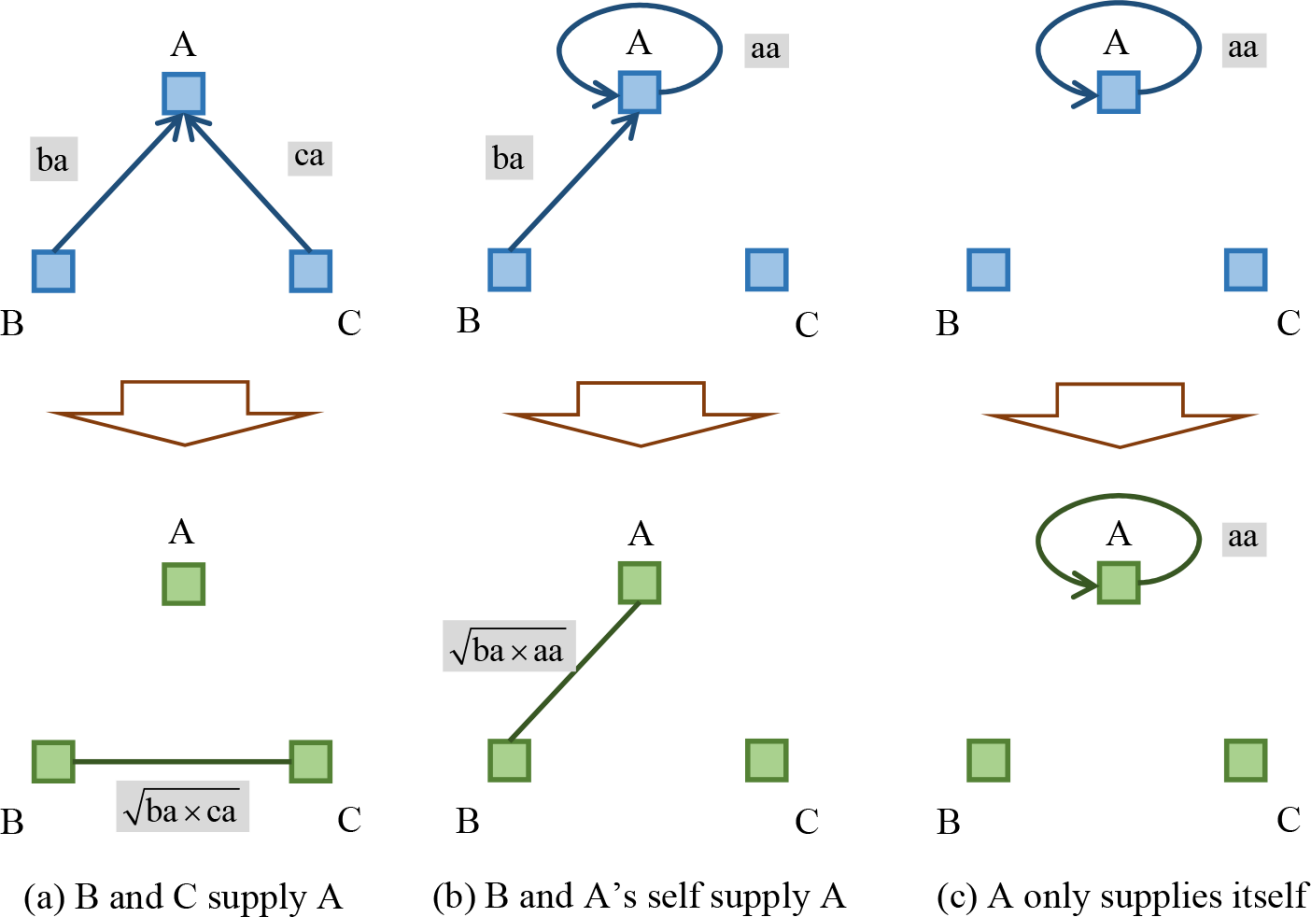
Three situations of transforming IO network with co-citation approach

There also exist three situations of inter-sector collaboration on providing resources together as shown in Fig 5. The computational process is basically the same as that of competition, but one thing here to note is that the direction of edge reflecting the flow of intermediate goods changes should be reverse.

## Modeling

There are two steps in modeling, the first is to extract the main economic relationships from intermediate inputs in IO table, and the second is to mine inter-sector competitive/collaborative relationships.

### Global industrial strongest relevant network model

In order to establish an industrial complex network, a sector is considered as a node, and the inter-industry IO relationship as an edge, whose weight represents the value of sale and purchase between producer and consumer. Thus, a graph *G* = (*V*,*E*,*W*) containing *n* nodes is created, representing sectors within a nation or region, as node set *V*. Pairs of nodes are linked by edges reflecting their dependencies, constituting an asymmetric edge set *E*. However, in valued graphs, set *E* is usually replaced by weight set *W*, which can be extracted from the quadrant I of IO table. Moreover, elements of main diagonal constitute nodes’ self-loops in graph *G*.

The table of intermediate inputs (the region of inter-country inter-industry) in WIOD2016R was straightforwardly adopted to build graph *G* in this paper, which actually is the aggregation of every single industrial value chain in the major economic entities. However, its topological structure needs to be further improved to solve empirical issues, such as ubiquitous competition/collaboration. But, graph *G* is too large to clearly reflect the reality of global economic system, and it must be refined according to a certain sort of threshold value.

In general network studies, distance between node *i* and node *j* is equal to the minimum number of edges connecting them, and we call a path whose length is equal to distance the **Shortest Path**, denoted by *d*_*ij*_. If there is no such path between node *i* and node *j*, *d*_*ij*_ = ∞. In non-weighted networks, the **Average Path Length (APL)** of the whole network can be calculated by Floyd algorithm [60], which measures the degree of separation of nodes. But, in weighted networks, especially when the similarity of nodes is proportional to their edge weight, it is infeasible to describe features of value stream flowing in economic system via APL. Thus, the classic Floyd algorithm must be revised, in order to make the new one capable of searching the path with the rapidest transfer and the lowest distortion of information, which is named the **Strongest Relevance Path Length (SRPL)**. The new iterative algorithm based on Floyd algorithm is:

$${\tilde{d}}_{ij}^{\left(k\right)}=\underset{i,j,k\in \left\{1,2,\ldots ,N\right\}}{\max}\left\{{\tilde{d}}_{ij}^{\left(k-1\right)},\frac{{\tilde{d}}_{ik}^{\left(k-1\right)}{\tilde{d}}_{kj}^{\left(k-1\right)}}{{\tilde{d}}_{ik}^{\left(k-1\right)}+{\tilde{d}}_{kj}^{\left(k-1\right)}}\right\}$$ (7)

where, ${\tilde{d}}_{ij}^{\left(k\right)}$ is SRPL between node *i* and node *j*, representing an industrial value chain on which each edge is the tightest and path is the shortest. According to Eq (7), one can get SRPLs between any two of nodes, and they are collected to form a new matrix ${\tilde{D}}^{\left(n\right)}$:

$${\tilde{D}}^{\left(n\right)}=\left(\begin{array}{ccc} {\tilde{d}}_{11}^{\left(n\right)} & \ldots & {\tilde{d}}_{1n}^{\left(n\right)}\\ \vdots & \ddots & \vdots \\ {\tilde{d}}_{n1}^{\left(n\right)} & \cdots & {\tilde{d}}_{nn}^{\left(n\right)} \end{array}\right)$$ (8)

The purpose of ${\tilde{D}}^{\left(n\right)}$ is to reflect the progressively enhanced economic relationships between nodes *i* and *j* through *n* intermediate sectors. Moreover, by comparison of ${\tilde{D}}^{\left(n\right)}$ and *W*, it is easy to notice the uniqueness that some SRPLs in the matrix is directly equal to their IO values. This phenomenon means that there exists both the strongest and most immediate industrial relevance in IO network. Thus, these elements could be extracted to form a new matrix, and the equations are:

$${\tilde{w}}_{ij}=\{\begin{array}{c} w_{ij},\;{\tilde{d}}_{ij}^{\left(n\right)}=w_{ij},\;i\neq j\\ w_{ij},\;{\tilde{d}}_{ij}^{\left(n\right)}\neq w_{ij},\;i=j\\ 0\;,\;{\tilde{d}}_{ij}^{\left(n\right)}\neq w_{ij},\;i\neq j \end{array}$$ (9)

**Fig 6:**
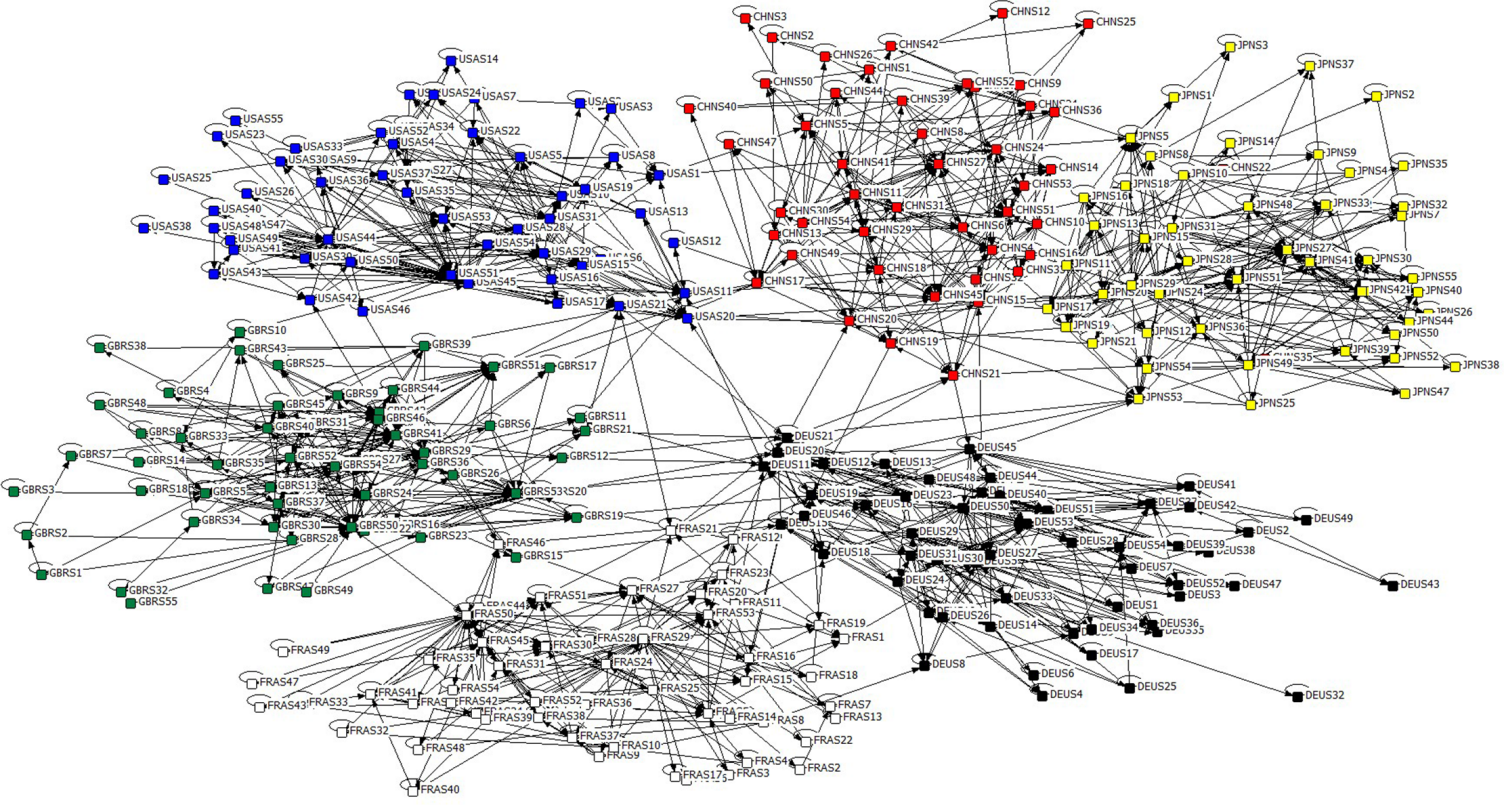
GISRN-WIOD2016R-2014 model.

Taking $\tilde{W}=\left\{{\tilde{w}}_{ij}\right\}$ as the adjacency matrix, **Global Industrial Strongest Relevant Network (GISRN)** can be established, which actually represents the crucial value stream flowing along the GVC (e.g., adjacency matrix of GISRN-WIOD2016R-2014 model is in S2 File). Self-loops in graph *G* remain, because the sector’s self-consumption ought to be as strongest and quickest as its SRPL with the others. Locally topological structure of GISRN-WIOD2016R-2011 is shown in Fig 6 after deleting weak industrial relevance via revised Floyd algorithm [61], including U.S., China, Japan, Germany, France and England (the GDP top 6 countries in 2014, but calculation are carried out on the basis of the whole network). Obviously, GISRN-WIOD2016R models belong to typical multiple complex network.

As mentioned in Table 1, WIOD2016R covers 43 independent economic entities and RoW (node’s name consists of the abbreviation of country/region and the sequence number of sector, e.g., CHNS12 respects China’s “Manufacture of basic pharmaceutical products and pharmaceutical preparations” sector), thus GISRN-WIOD2016R models virtually illustrate the major component of inter-sector economic relationships on the GVC. It is clear that there are more linkages existing within one economic entity than across boundaries. These models make it possible to observe how main value stream flows along the GVC.

Although GISRN models describe the main industrial relevance around the world, the hidden information about their inter-sector competition/collaboration also needs to be further revealed by data mining.

### Global industrial resource competition network model

Generally speaking, inter-sector multiple competition is equal to the sum of geometric mean of single competition, whether happens between different sectors or inside themselves. **Global Industrial Resource Competition Network (GIRCN)** is therefore established based on three types of competition for production resources, and its adjacency matrix $\vec{W}=\left\{{\vec{w}}_{ij}\right\}$ generation algorithms are as follows:

$${\vec{w}}_{ij}=\{\begin{array}{c} \sum_{k=1}^{n}\sqrt{{\tilde{w}}_{ki}{\tilde{w}}_{kj}},i\neq j\\ \;{\tilde{w}}_{ij}\;,i=j \end{array}$$ (10)

where, ${\vec{w}}_{ij}$ is the i-th row and j-th column element in the adjacency matrix (also edge weight matrix) of GIRCN models, which measures the accumulative quantitative degree coming from competition between node *i* and node *j* (adjacency matrix of GIRCN-WIOD2016R-2014 model is in S3 File).

**Fig 7:**
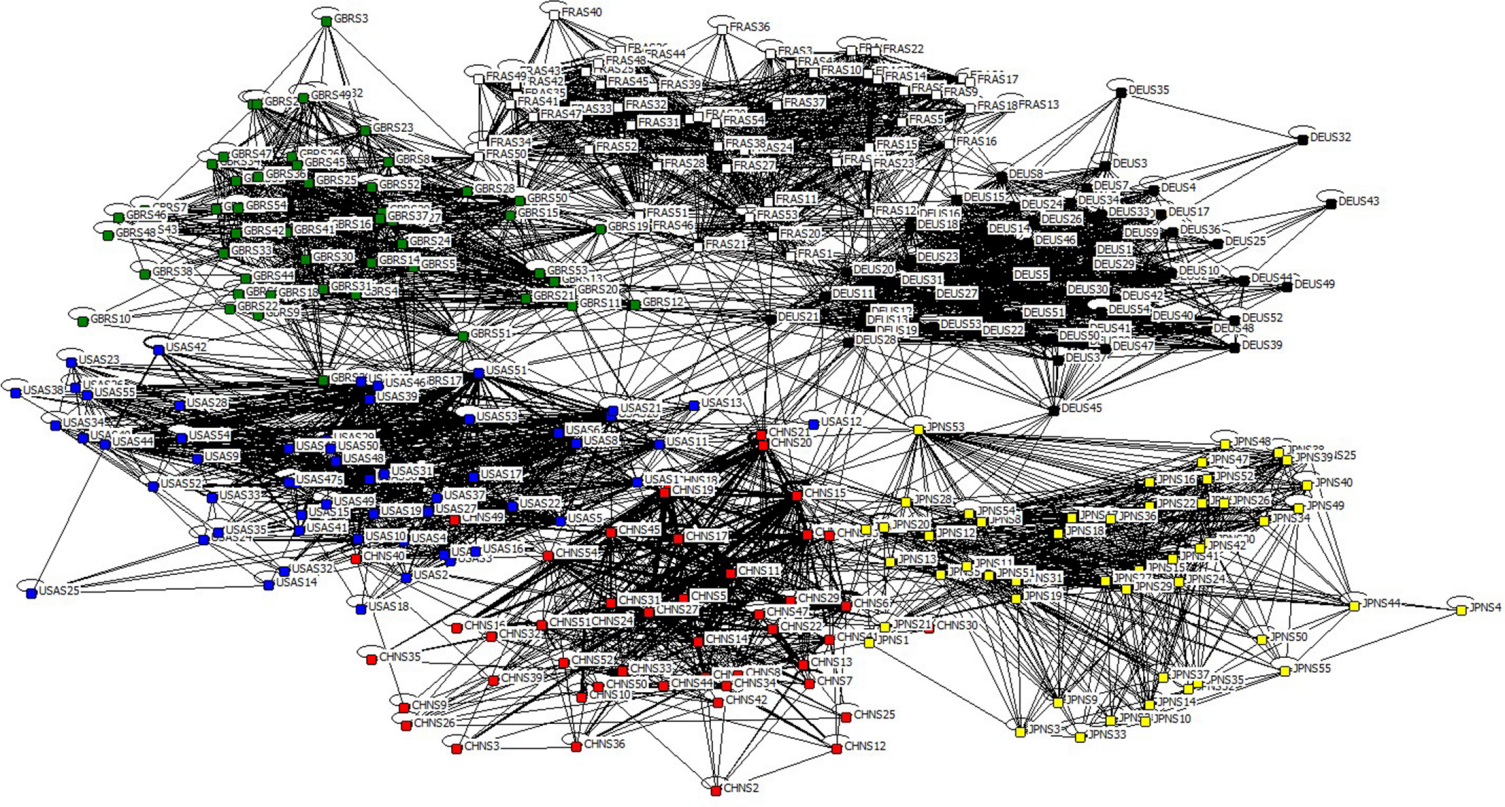
GIRCN-WIOD2016R-2014 model.

Because the inter-sector relationship is defined from an objective perspective (not with the intuitive reflect of WIOD), the degree of competition is even for both sides, i.e., $\vec{W}=\left\{{\vec{w}}_{ij}\right\}$ is symmetric according to Eq (10). Locally topological structure of GIRCN-WIOD2016R-2014 is shown in Fig 7, where edges’ thickness is proportional to their edge weights.

Contrast to previous studies, the most significant feature of GIRCN models is that it’s a weighted but non-directed network, because the competition between industry sectors is mutual, i.e., there is no need to use the edge’s direction to show who is the initiator or receiver of the competition. In addition, nodes’ self-loops in GIRCN models embody not only the fact that sectors contest with the others for products and services in global economic system, but also that they consume what they have produced. In other words, relative to the others, each of sectors is also likely to be its own competitor.

### Global industrial production collaboration network model

**Fig 8:**
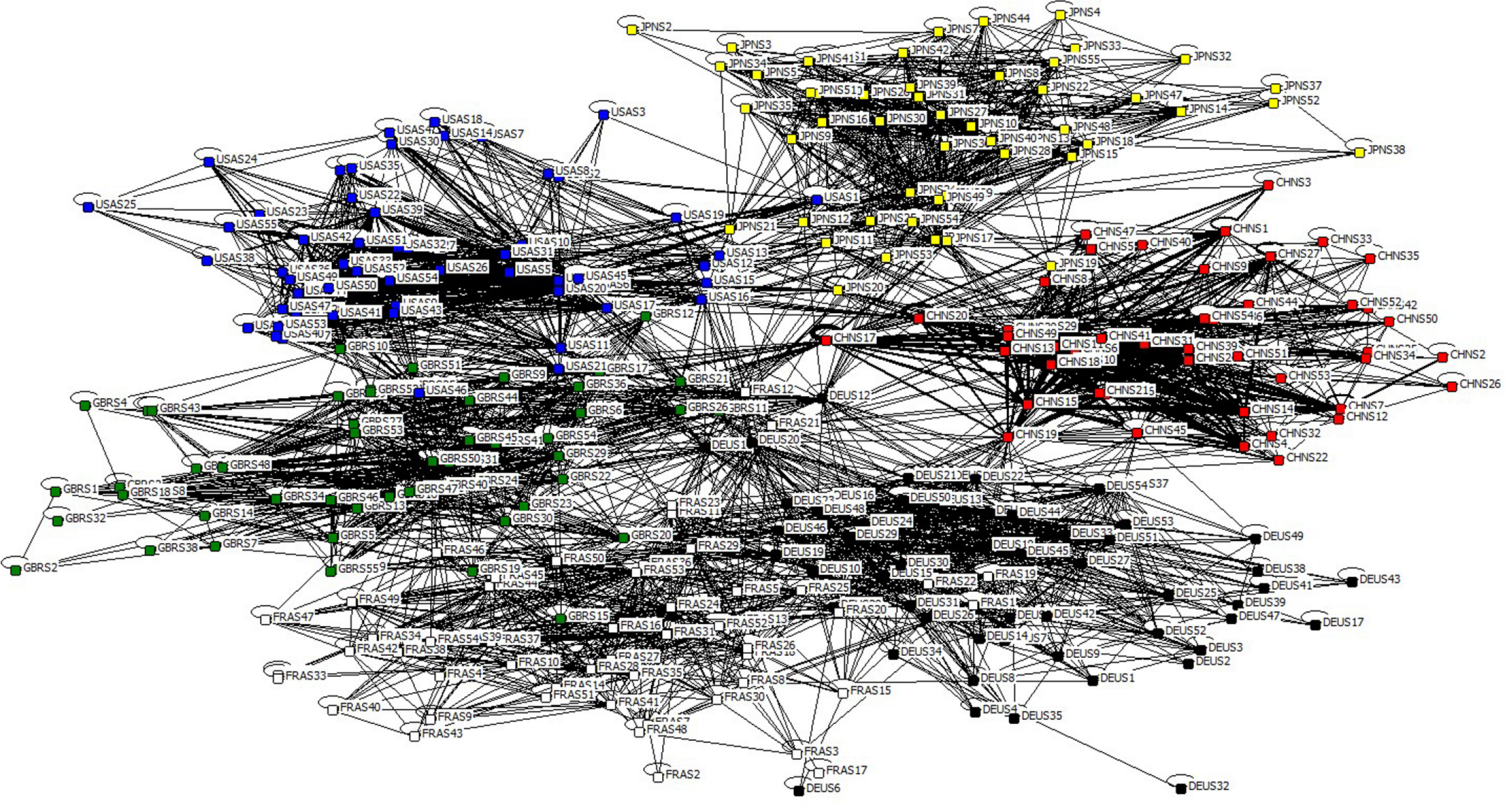
GIPCN-WIOD2016R-2014 model.

In order to reproduce the collaborative relationship between industrial sectors on the GVC, co-citation approach is here introduced to establish an industrial complex network, which is named **Global Industrial Production Collaboration Network (GIPCN)**. Similar with GIRCN models, there are also three types of collaboration on industry development in GIPCN, and its adjacency matrix $\overset{\leftarrow }{W}=\left\{{\overset{\leftarrow }{w}}_{ij}\right\}$ generation algorithms are as follows:

$${\overset{\leftarrow }{w}}_{ij}=\{\begin{array}{c} \sum_{k=1}^{n}\sqrt{{\tilde{w}}_{ik}{\tilde{w}}_{jk}},i\neq j\\ \;{\tilde{w}}_{ij}\;,i=j \end{array}$$ (11)

where, ${\overset{\leftarrow }{w}}_{ij}$ is the i-th row and j-th column element of adjacency matrix of GIPCN models, which measures the accumulative quantitative degree coming from collaboration between nodes *i* and *j* (adjacency matrix of GIPCN-WIOD2016R-2014 model is in S4 File). Locally topological structure of GIPCN-WIOD2016R-2014 is shown in Fig 8.

Although there is a visual similarity between GIPCN model and GIRCN model, the sectors out of the largest connected component in these two models are actually different. This is because that some nodes locate on tail ends as sink nodes in GIRCN models, but they may change the roles to be source nodes in GIPCN models, therefore the edge forming mechanism is distinctive to these two kinds of derivative models.

In sum, the purpose of GISRN model is to depict the main value flow on the GVC, as well as reduce the influence on analysis out of the subordinate IO relationships. GIRCN/GIPCN models based on bibliographic coupling/co-citation approach are established to abstract the competitive/collaborative status hidden in IO tables. Thus, concrete complex network indicators are needed to analyze the structural features of the GVC in next.

## Measurement

In common IO analysis, the sum of output or input of a given industrial sector, equivalently the out-strength or in-strength of a node in GIVCN model [28], is used to quantify the value streams before or after flowing through the node. It cannot be used directly to describe the inter-sector relationships, nor its impact on the whole economic system. Therefore, modeling solutions from citation network are here adopted to realize the competitive/collaborative relationships between each pair of sectors, and then network-based indicators are introduced to reveal how sectors exactly compete for limited production resources from their mutual upstream providers, and collaborate to maintain the development of industrial system on the basis of GIRCN/GIPCN models.

### Unit weight

**Fig 9:**
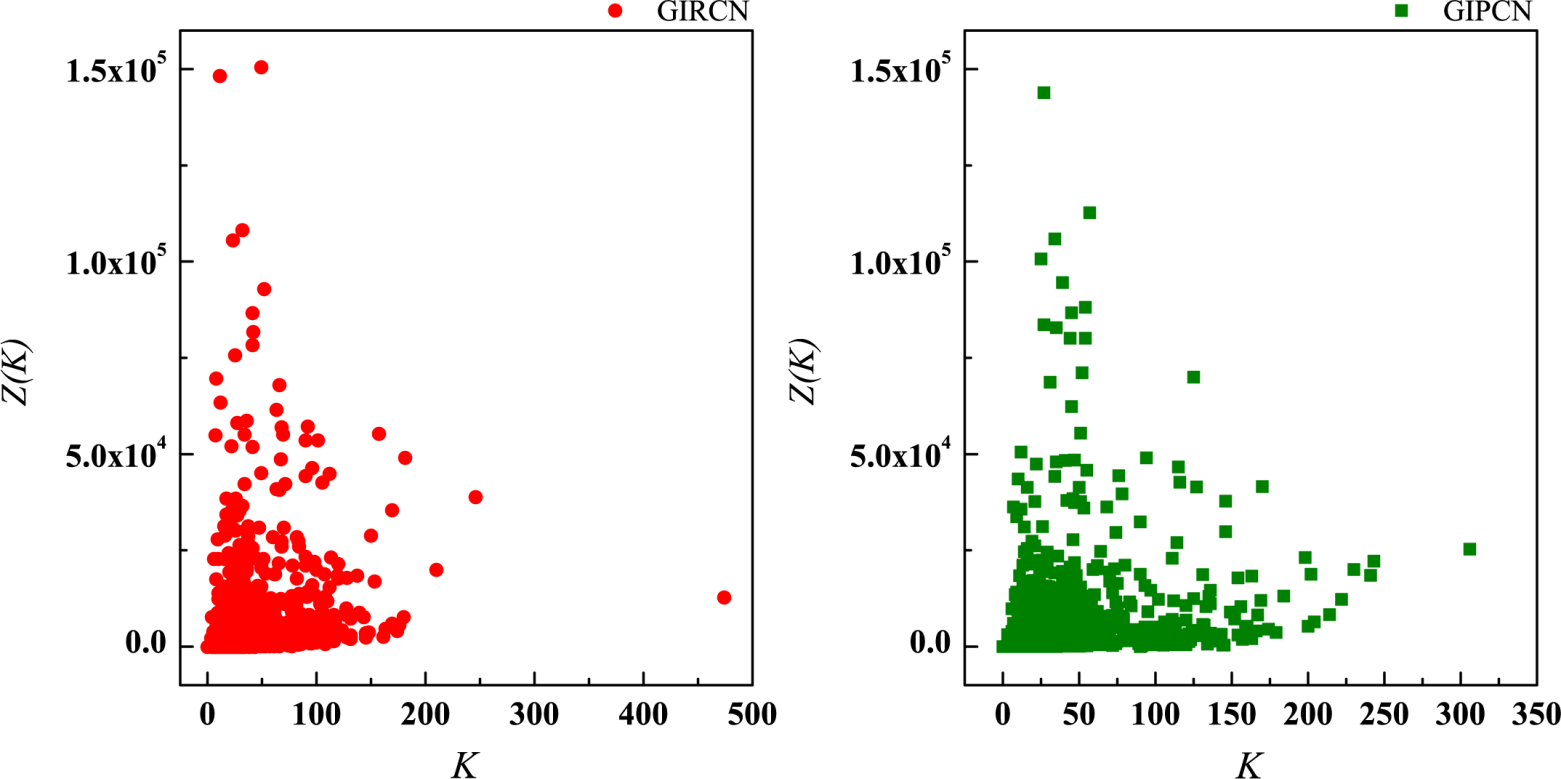
Correlation between degree and unit weight.

**Fig 10:**
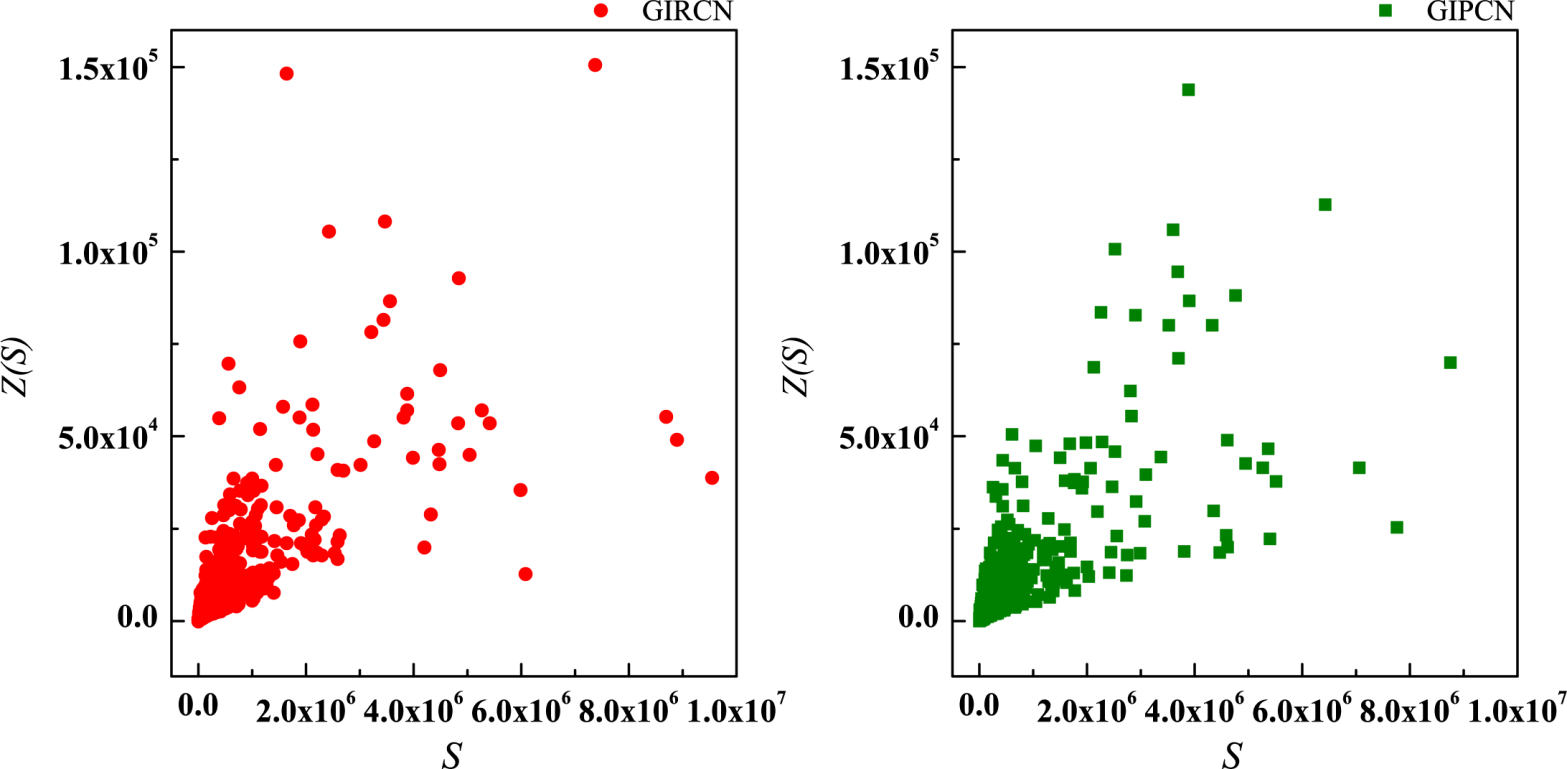
Correlation between strength and unit weight.

**Unit Weight** *Z*(*i*) is a description of the average level of node strength, which is usually used to measure the social inertia of research object in social network analysis. For instance, when studying scientific collaboration network, some of scientists join in a lot of research team but with little contribution, so they own small unit weights in the network. Otherwise, preferring to cooperate with rare people and doing in-depth research will bring them large unit weights [62]. The definition of unit weight is the ratio of node’s strength to degree, which is similar in essence to expectation in statistics and defined as **Competitive/Collaborative Power** in this paper. Its equation is as follows:

$$Z\left(i\right)=\frac{S\left(i\right)}{K\left(i\right)}=\frac{\sum_{j=1}^{n}{\vec{w}}_{ij}}{K\left(i\right)}$$ (12)

where, *K*(*i*) is the degree of node *i*, and *S*(*i*) is the strength of node *i*. In Figs 9 and 10, correlations between *K*(*i*) and *Z*(*i*), *S*(*i*) and *Z*(*i*) are analyzed respectively (all OriginPro graphs of correlation analyses in this paper are in S5 File).

The correlation results are both low, which means unit weight has nothing to do with the number of connection and the strength of node.

In terms of the mathematics, *Z*(*i*) is the average of the edge weights of a certain node in GIRCN/GIPCN models. But, in terms of economics, there is a positive correlation between node’s unit weight and sector’s competitive/collaborative power in global economic system. In details, sectors with high *Z*(*i*) simultaneously compete for production resource with the others on the GVC in GIRCN models, or jointly provide intermediate goods to the same consumers in GIPCN models. By any means, these sectors take an irreplaceable position in the aspect of maintaining trade activities.

Top 10 sectors of *Z*(*i*) in both GIRCN-WIOD2016R and GIPCN-WIOD2016R models from 2000 to 2014 (every five years) are shown in Tables 2 and 3 (all the statistical results of indicators in GIRCN-WIOD2016R models are in S6 File, and those of GIPCN-WIOD2016R models are in S7 File).

**Table 2:** Top 10 sectors of unit weight in GIRCN.

Rank	2000	2004	2009	2014
1	USAS41	USAS44	CHNS27	CHNS27
2	USAS44	USAS36	USAS44	CHNS16
3	USAS50	USAS30	CHNS16	CHNS14
4	USAS29	USAS53	CHNS14	CHNS1
5	USAS30	USAS50	CHNS1	CHNS18
6	USAS36	USAS29	CHNS18	CHNS6
7	USAS53	USAS41	USAS53	CHNS4
8	USAS45	USAS45	USAS36	USAS44
9	USAS42	USAS43	CHNS4	CHNS13
10	USAS51	USAS5	USAS42	CHNS12

**Table 3:** Top 10 Sectors of unit weight in GIPCN.

Rank	2000	2004	2009	2014
1	USAS44	USAS44	USAS44	CHNS4
2	USAS41	USAS41	CHNS4	CHNS11
3	USAS39	USAS42	CHNS24	CHNS24
4	USAS51	USAS45	USAS42	CHNS1
5	USAS42	USAS39	CHNS1	CHNS16
6	USAS45	USAS5	CHNS11	USAS44
7	USAS40	USAS51	CHNS16	CHNS10
8	USAS5	USAS40	CHNS19	CHNS7
9	JPNS16	USAS4	USAS41	CHNS5
10	JPNS49	CHNS1	CHNS18	CHNS41

Among the globally competitive relationships, U.S. industrial sectors occupy all of the top 10 positions in 2000 and 2004, showing great competitive power in international trade. But in 2014, only one of them remain in the top 10 of the list, with all the others changed to Chinese sectors. It is easy to find that their competitive powers are gradually shrinking ever since. Taking USAS44 “Real estate activities” and USAS41 “Financial service activities, except insurance and pension funding" as examples, they were in the very top of the list, but fell significantly in the ranking after 2009, which is the year subprime crisis began to affect worldwide financial markets.

On the contrary, Chinese "Construction" (CHNS27) and manufacturing sectors, such as "Manufacture of other non-metallic mineral products" (CHNS14), "Manufacture of fabricated metal products, except machinery and equipment" (CHNS16) and "Manufacture of electrical equipment" (CHNS18), overtook American sectors as the most powerful ones on GVC around 2009, attracting and consuming lots of resources. This pretty clear trend could explain why China's economy has advanced rapidly growing into a leading power during that period.

From the perspective of collaboration, U.S. occupied the top 8 of the list in 2000, but only one of them remain in the top 10 of the list, with all the others also changed to Chinese sectors in 2014. American sectors of "Financial service activities, except insurance and pension funding" (USAS41), "Insurance, reinsurance and pension funding, except compulsory social security" (USAS42) and "Real estate activities" (USAS44) used to be good at promoting the local even global economic development, which all belong to the tertiary industry. However, these sectors were replaced by some of Chinese primary industry and basic industry around 2009, such as "Crop and animal production, hunting and related service activities" (CHNS1) and "Mining and quarrying" (CHNS4) for the former, or "Manufacture of chemicals and chemical products" (SCHN11), "Manufacture of fabricated metal products, except machinery and equipment" (CHNS16) and "Electricity, gas, steam and air conditioning supply" (CHNS24) for the latter. Rich natural resources and cheap labor resources are the major driving force for these Chinese sectors to enhance the collaborative power with the others on the GVC.

### Disparity in the weight

**Disparity in the Weight** *Y*(*i*) describes the dispersion degree of edge weights related to node *i* [63]. It was here introduced to measure the disequilibrium of competition or collaboration in global economic system, which is defined as **Competitive/Collaborative Amplitude** in this paper. Its equation is as follows:

$$Y\left(i\right)=\sum_{j=1}^{n}{\left[\frac{{\vec{w}}_{ij}}{S\left(i\right)}\right]}^{2}=\frac{\sum_{j=1}^{n}{\left({\vec{w}}_{ij}\right)}^{2}}{{\left(\sum_{j=1}^{n}{\vec{w}}_{ij}\right)}^{2}}$$ (13)

**Fig 11:**
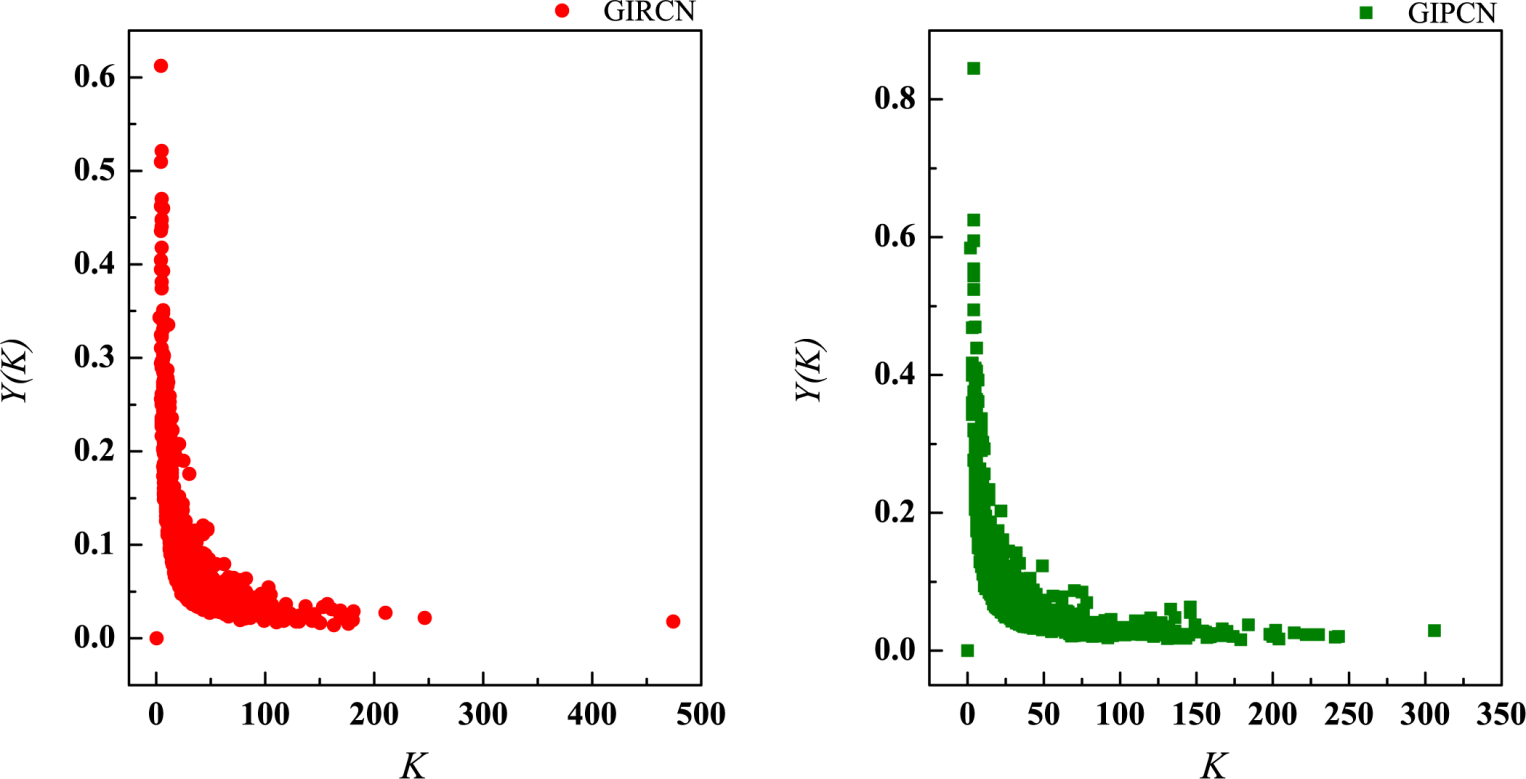
Correlation between degree and disparity in the weight.

**Fig 12:**
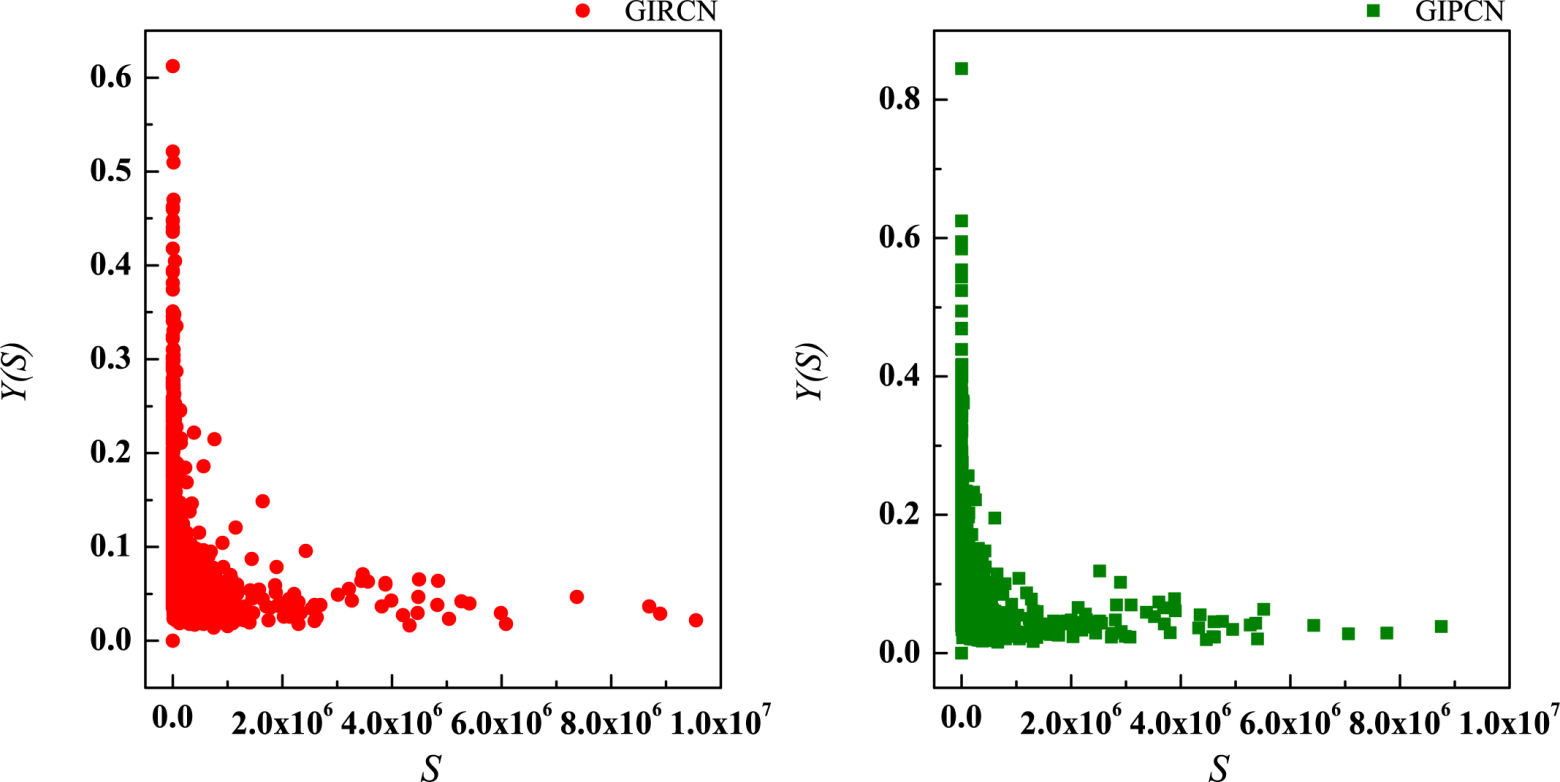
Correlation between strength and disparity in the weight.

**Fig 13:**
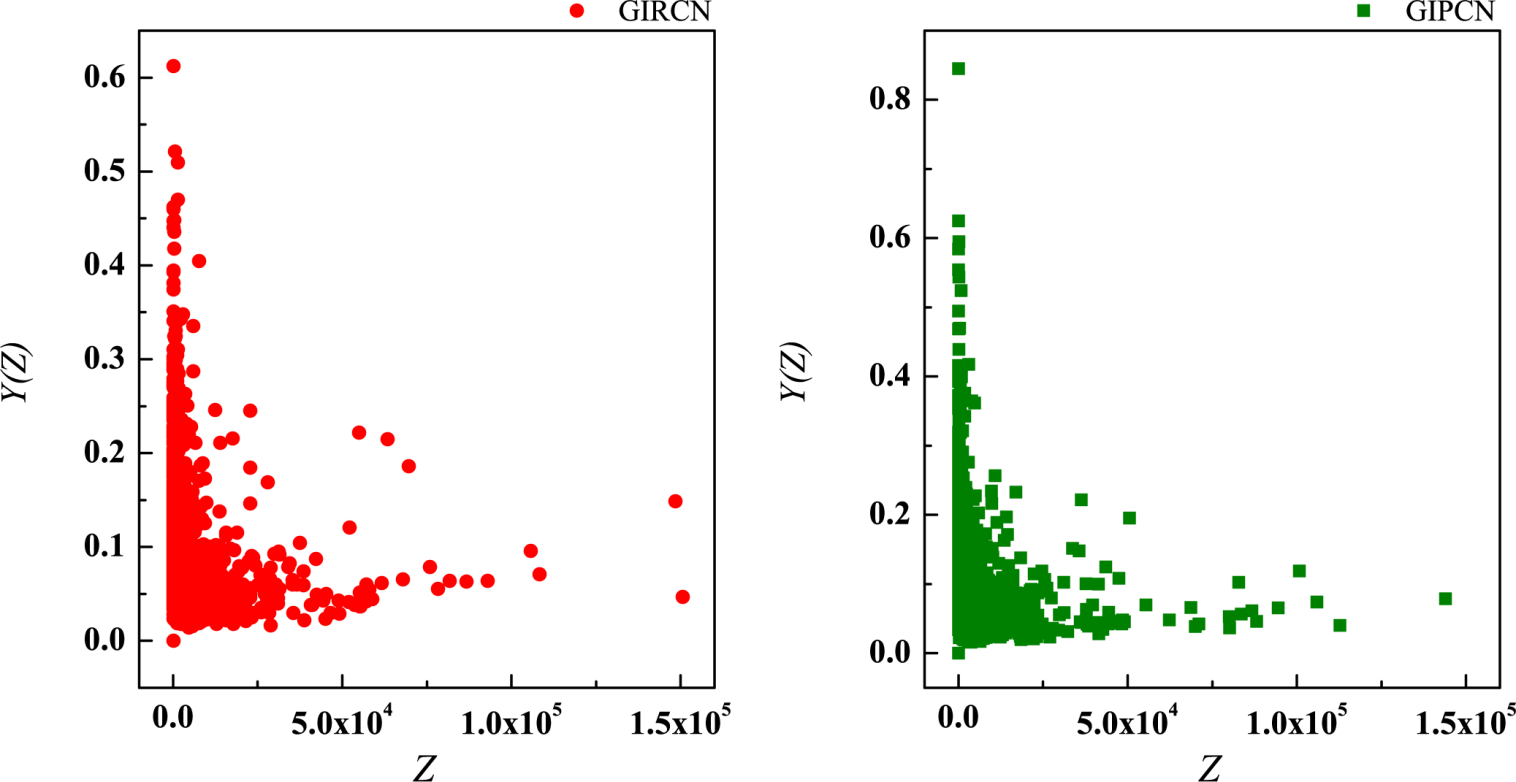
Correlation between unit weight and disparity in the weight.

Although there is no *K*(*i*) in Eq (13), actually equals to the number of ${\vec{w}}_{ij}$ linked to node *i* in GIRCN/GIPCN models. Therefore, correlation analyses are carried out between *K*(*i*), *S*(*i*), *Z*(*i*) and *Y*(*i*) in Figs 11–13.

It is clear that, *Y*(*i*) experiences steep decline with rising of *K*(*i*) in the right part of the curve in Fig 11, and then turns to slow down. However, correlation between *S*(*i*) and *Y*(*i*) is non-significant, as well as *Z*(*i*) and *Y*(*i*). The results suggest that, the higher *K*(*i*) node *i* has, the lower *Y*(*i*) it gets. As for the phenomena of corner point during descending, it is about the nature of GISRN models.

There are two types of trade incorporated in GISRN models: international trade and domestic trade. Correspondingly, competition/collaboration exists on two levels in GIRCN/GIPCN models, i.e., within homeland (or economic entity) and cross border. For most of sectors, they obtain raw materials or intermediate products interiorly. Thus, the number of their relevant sectors is reasonably less than 56, which is the number of classifications in WIOD. As globalization becomes prevalent, some of them generalize procurement all over the world, which brings them more connections with exotic sectors. Thus, the corner points can be treated as the blurred boundary of industrial globalization.

High *Y*(*i*) means that the majority of competition/collaboration that sector *i* engages in has a great difference, i.e., sector *i* obtains its production resources or convey its outcomes via a relatively narrow channel. In fact, there is huge gap between its inner and outer competitive/collaborative powers on the GVC. Otherwise, sector with low *Y*(*i*) tends to be competitive/collaborative similarly in both international and domestic markets. Thus, there is a negative correlation between node’s disparity in the weight and sector’s competitive/collaborative amplitude.

In consideration of the real economic activities, fierce competition or close collaboration usually happens in the same economic entity in most cases, so higher *Y*(*i*) also corresponds to lower degree of conditional globalization that may be cross-border, and vice versa.

Top 10 sectors of *Y*(*i*) in both GIRCN-WIOD2016R and GIPCN-WIOD2016R models from 2000 to 2014 (every five years) are shown in Tables 4 and 5.

**Table 4:** Top 10 sectors of disparity in the weight in GIRCN.

Rank	2000	2004	2009	2014
1	TURS47	LUXS20	CYPS18	CYPS41
2	TWNS28	TWNS28	TWNS28	TWNS28
3	INDS32	CYPS18	CYPS41	KORS2
4	LVAS11	LUXS2	LUXS42	LUXS42
5	BGRS3	HRVS48	KORS2	BGRS3
6	CYPS49	INDS32	IRLS6	LUXS38
7	CYPS18	CYPS41	TURS26	IRLS25
8	ESTS10	LVAS11	MLTS42	MLTS10
9	LUXS2	BGRS3	BGRS3	IDNS35
10	CHES2	IRLS6	ESPS1	BELS25

**Table 5:** Top 10 sectors of disparity in the weight in GIPCN.

Rank	2000	2004	2009	2014
1	NLDS2	NLDS2	TWNS2	CYPS10
2	TWNS55	TWNS55	HUNS3	MLTS47
3	LUXS4	TWNS2	IDNS25	IDNS25
4	HUNS3	HUNS3	SVKS32	MLTS2
5	TURS3	SVNS47	CYPS21	LUXS26
6	JPNS37	TURS3	GRCS2	TWNS2
7	KORS4	CYPS15	LTUS4	PRTS4
8	BGRS43	ESPS43	ESPS43	BGRS26
9	ITAS3	GRCS2	BGRS43	TURS3
10	TWNS4	SVNS33	BELS2	MLTS7

Statistically, sectors with less competitive amplitude are confined to the small economies such as Cyprus, Luxembourg, Chinese Taipei, Latvia and Ireland. These sectors mainly compete for civil or regional production resources rather than intermediate inputs on the global market. In other words, these sectors just compete with few others within the same region. It is clear that some sectors locate at the ends of branches in the GISRN models, thus their competitive amplitudes are extremely narrow, even though there are no values for their *Y*(*i*).

In comparison with the situation in GIRCN models, sectors with less collaborative amplitude are also confined to the small economies. Anyway, these sectors basically serve for domestic economic development, which means their process of globalization has been lagging behind.

### Weighted clustering coefficient

Clustering Coefficient is an important gauge of network collectivization, and measures the degree to which nodes in a network tend to cling together. In particular social networks, nodes tend to create tightly knit groups characterized by a relatively high density of edges [64]. This indicator has profound social meaning, because the phenomenon of cluster prevails in many social networks. But, in weighted complex network, it should be modified to take edge weights into consideration. Hence, **Weighted Clustering Coefficient** *C*^*W*^(*i*) is here introduced to reflect the degree of competitive/collaborative involvement of sector *i* on the GVC.

First and foremost, global clustering coefficient is adopted as the basis of modification [65], for *C*^*W*^(*i*) has been used to measure inter-sector competition/collaboration on GVC. It is known that the global clustering coefficient is based on triplets of nodes. For simplicity, it is the number of closed triplets (which consist of three connected nodes) over the total number of triplets (both open and closed). Therefore, we just need to consider how to substitute edge weight matrix with binary adjacency matrix when carrying out matrix transformation. One way is as follows (the equation is directly given without derivation process, please see my team’s early research for details [66]):

$$C^{W}\left(i\right)=\frac{\sum_{j=1}^{n}\sum_{k=1}^{n}{\left({\vec{w}}_{ij}{\vec{w}}_{jk}{\vec{w}}_{ki}\right)}^{\frac{1}{3}}}{K\left(i\right)\left(K\left(i\right)-1\right)}$$ (14)

**Fig 14:**
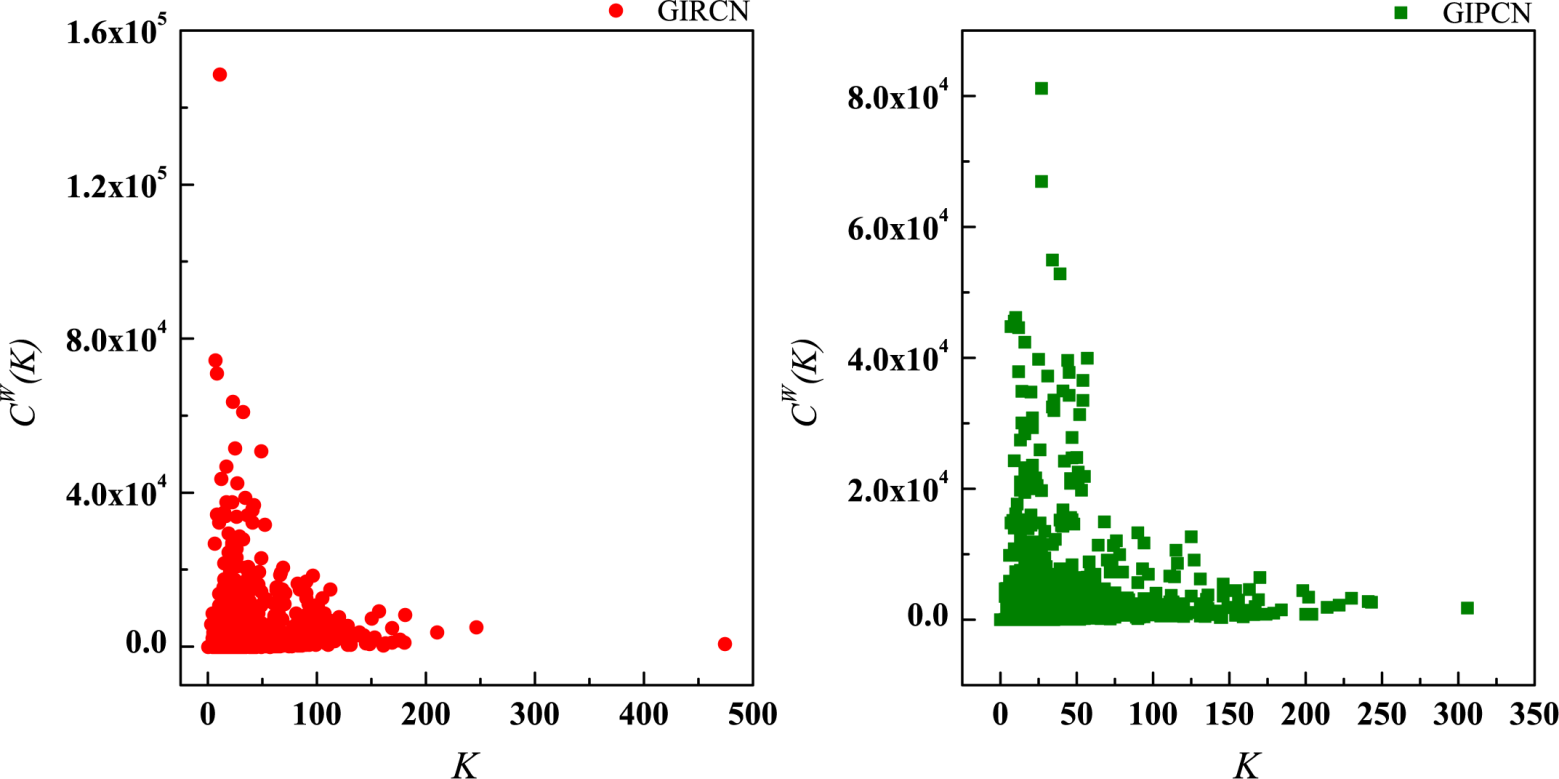
Correlation between degree and weighted clustering coefficient.

**Fig 15:**
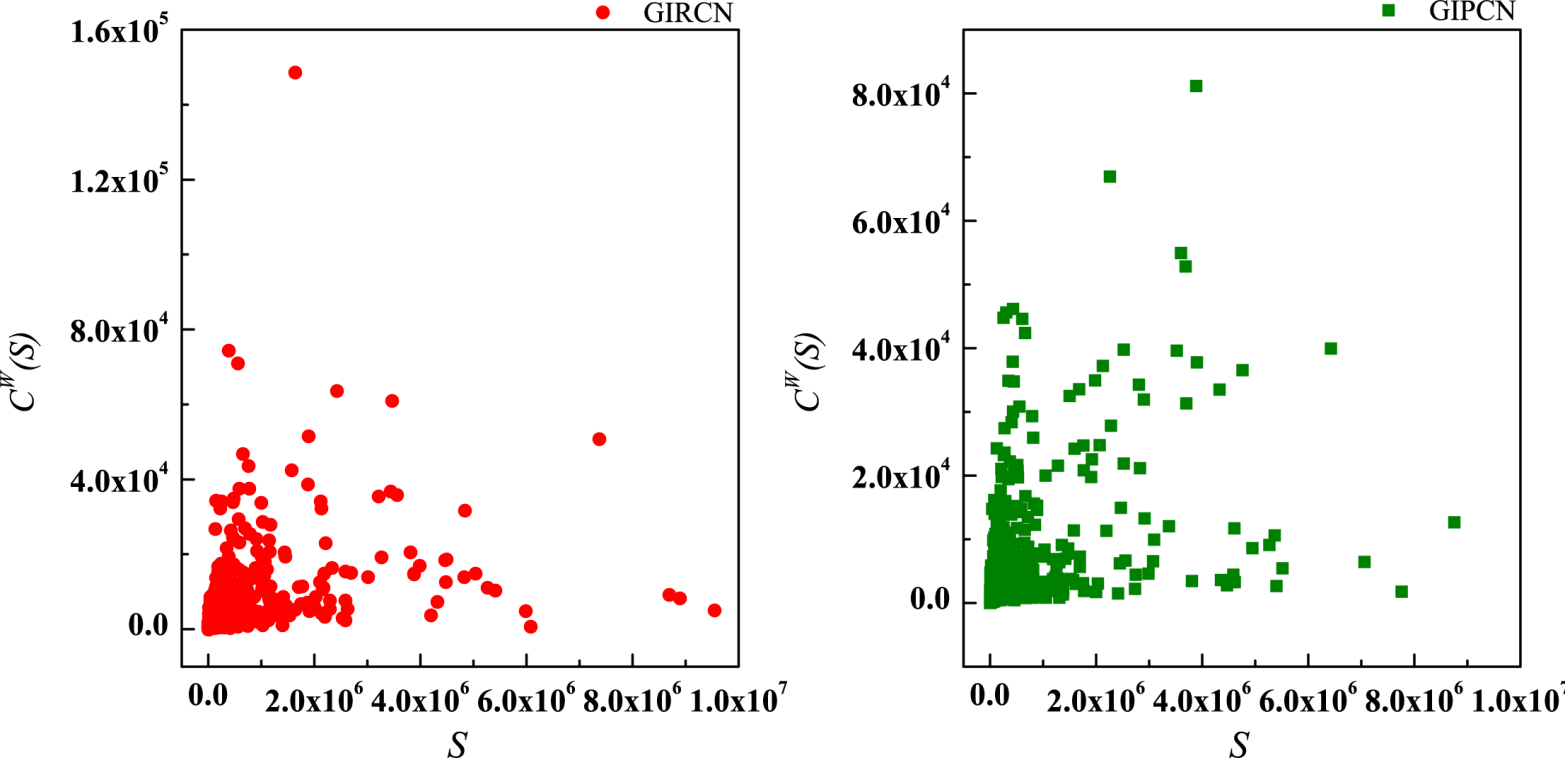
Correlation between strength and weighted clustering coefficient.

**Fig 16:**
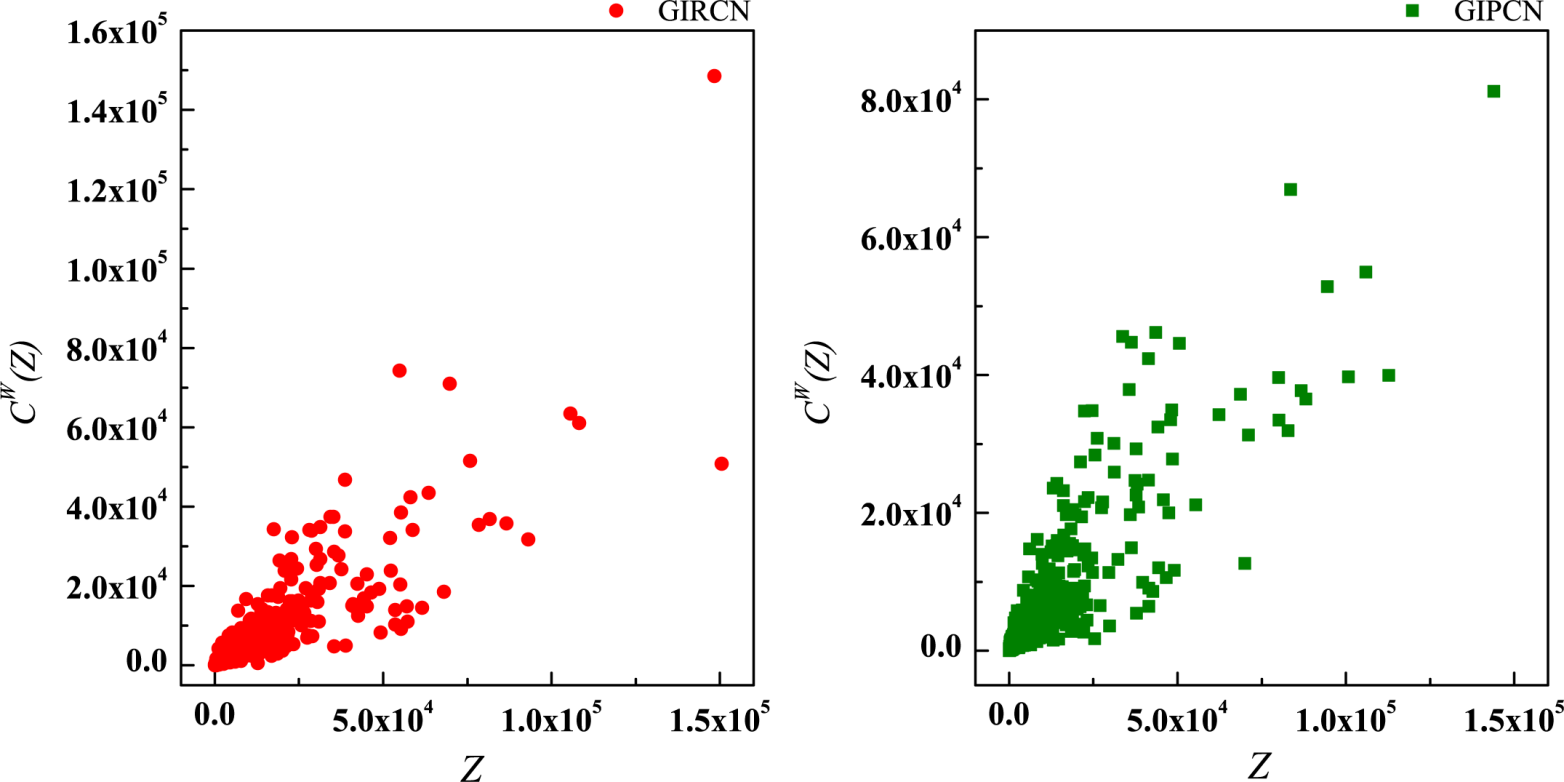
Correlation between unit weight and weighted clustering coefficient.

**Fig 17:**
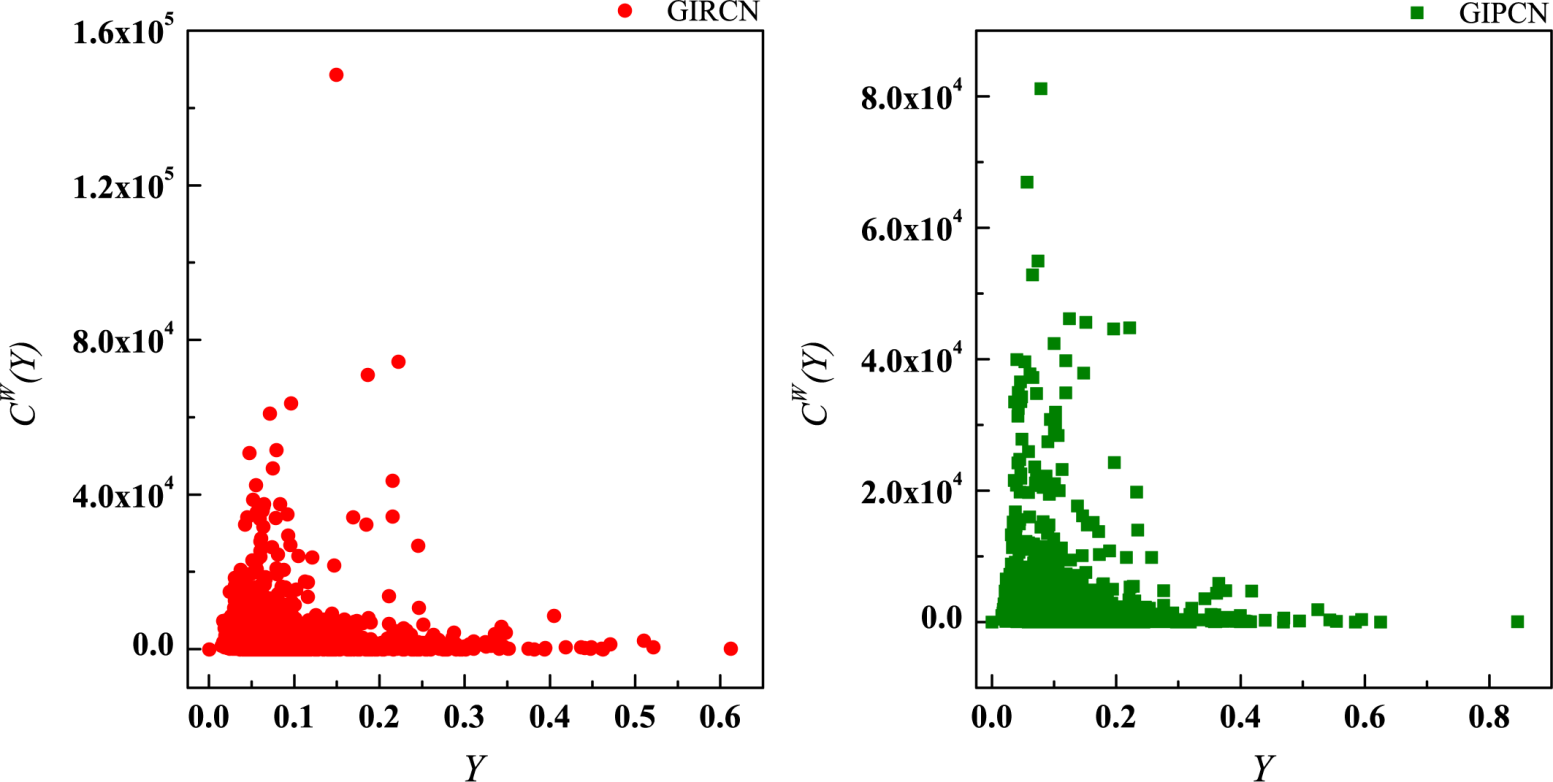
Correlation between disparity in the weight and weighted clustering coefficient.

According to Eq (14), sector with high *C*^*W*^(*i*) means that it has been involved in both tight and closed competitive/collaborative environments, i.e., competition/collaboration not only exists between one sector and its counterparts but also prevails among its counterparts at the same time. Actually, *C*^*W*^(*i*) measures the degree of industrial clustering from the perspective of competition/collaboration, which is defined as **Competitive/Collaborative Intensity** in this paper. Therefore, in GIRCN/GIPCN models, there is a positive correlation between node’s weighted clustering coefficient and sector’s competitive/collaborative intensity. Correlation analyses are carried out between *K*(*i*), *S*(*i*), *Z*(*i*), *Y*(*i*) and *C*^*W*^(*i*) in Figs 14–17.

Obviously, *Z*(*i*) can better explains *C*^*W*^(*i*) than the other measurements (no matter whether with weight or not), i.e., only sector’s competitive/collaborative power has significant effect on its competitive/collaborative intensity.

There is no doubt that, a certain degree of ordered competition/collaboration can improve the operation efficiency of industrial system, so sectors with higher competitive/collaborative intensity effectively will lead the economic development within relevant the industrial clusters.

10 sectors with top *C*^*W*^(*i*) in both GIRCN-WIOD2016R and GIPCN-WIOD2016R models from 2000 to 2014 (every five years) are shown in Tables 6 and 7.

**Table 6:** Top 10 sectors of weighted clustering coefficient in GIRCN.

Rank	2000	2004	2009	2014
1	USAS38	USAS36	CHNS16	CHNS16
2	USAS41	USAS52	CHNS3	CHNS3
3	USAS36	USAS30	CHNS14	CHNS12
4	CHNS16	USAS44	USAS52	CHNS1
5	USAS52	USAS38	USAS44	CHNS14
6	JPNS49	USAS54	CHNS12	CHNS13
7	JPNS44	CHNS16	USAS36	CHNS27
8	USAS30	USAS50	CHNS13	CHNS8
9	CHNS3	JPNS44	CHNS27	CHNS36
10	JPNS39	USAS41	CHNS7	CHNS31

**Table 7:** Top 10 sectors of weighted clustering coefficient in GIPCN.

Rank	2000	2004	2009	2014
1	USAS41	USAS35	CHNS4	CHNS4
2	USAS35	USAS41	CHNS24	CHNS7
3	USAS44	USAS44	CHNS7	CHNS24
4	USAS40	USAS42	CHNS3	CHNS16
5	USAS39	JPNS19	USAS44	CHNS12
6	JPNS1	USAS39	USAS40	USAS38
7	USAS51	USAS40	USAS42	CHNS2
8	USAS42	USAS51	CHNS16	CHNS3
9	USAS38	JPNS1	USAS39	USAS34
10	JPNS14	USAS36	USAS41	CHNS11

As for statistics on competitive intensity, there are two significant differences between U.S. and China. On one hand, U.S. sectors with high *C*^*W*^(*i*) once occupied the top of rank, such as "Retail trade, except of motor vehicles and motorcycles"(USAS30), "Accommodation and food service activities" (USAS36) and "Motion picture, video and television programme production, sound recording and music publishing activities; programming and broadcasting activities" (USAS38), which fall into the category of service industry. On the other hand, Chinese sectors went beyond those of U.S. step by step to become the core of industrial clusters, and they cover nearly all the important sectors of industry and agriculture, which indicates that these two countries have experienced different courses of economic development.

Sectors owning higher collaborative intensity show that industrial clusters with complete internal structure have already formed around them, and they are able to optimize resource collocation and enhance industrial competitiveness by providing indispensable products or services. It can be seen in Table 7 that China' s economic development has concrete grounds on natural resources and energy, involving "Mining and quarrying" (CHNS4), "Manufacture of wood and of products of wood and cork, except furniture; manufacture of articles of straw and plaiting materials"(CHNS7) and "Electricity, gas, steam and air conditioning supply"(CHNS24).

## Conclusions

The ongoing economic globalization has pushed the industrial value chains of various nations into a connected one, forming the global industrial value chain network. Only with solid assessment on countries’ functions and status under the framework of world economic system, can correct and optimal decisions be made for strategic positioning, routine planning and policy making, so as to reach the targets of expanding marketing shares and strengthening competitive advantages.

Both bibliographic coupling and co-citation approaches in citation network theory have been technically chosen for network modeling and data mining. Refinement on these approaches has been accomplished to set up GIRCN/GIPCN models describing the main inter-sector competitive/collaborative relationships on the GVC. Statistical indicators suitable for such weighted and non-directed network with self-loops are thereafter introduced to quantify sectors’ properties under globalization, including unit weight, disparity in the weight and weighted clustering coefficient. These three indicators represent distinguishing social network meanings, which are respectively inertia, disequilibrium and collectivization of nodes. Whether there are dependency between any two of them or to what extent they are quantitatively similar are entirely up to the heterogeneity of network structure. Theoretical derivation and empirical study shows that a positive correlation exists between unit weight and competitive/collaborative power, and it is also the case between weighted clustering coefficient and competitive/collaborative intensity, whereas a negative correlation between disparity in the weight and competitive/collaborative amplitude.

The proposed research framework in this paper has made it possible to evaluate and scale the functions and competitions of economic entities, countries and industrial sectors in the global economic system. Evolution mechanism of industrial specialization pattern and its influence on upgrading domestic industrial structure can thus be revealed. Furthermore, simulations and scenario analysis on the functions, positions and countermeasures of key nations and industrial sectors on the global industrial value chain network are to be carried out under the prospective of economic globalization so as to bring about constructive suggestions on promotion of optimal allocation of global industrial resources and upgrading of international competitiveness.

## Supporting information

S1 File Names and abbreviations of countries and sectors in WIOD2016R. (XLSX)

S2 File Adjacency matrix of GISRN-WIOD2016R-2014 model. (XLSX)

S3 File Adjacency matrix of GIRCN-WIOD2016R-2014 model. (XLSX)

S4 File Adjacency matrix of GIPCN-WIOD2016R-2014 model. (XLSX)

S5 File OriginPro graphs of correlation analyses. (OPJ)

S6 File Statistical results of indicators in GIRCN-WIOD2016R models. (XLSX)

S7 File Statistical results of indicators in GIPCN-WIOD2016R models. (XLSX)
